# Pyramiding Recessive Resistance Genes Enhances Bacterial Leaf Spot Resistance in Peppers by Suppressing *In Planta* Bacterial Growth

**DOI:** 10.3390/plants14162559

**Published:** 2025-08-17

**Authors:** Mousami Poudel, Sophia McDuffee, Gerald V. Minsavage, Samuel F. Hutton, Anuj Sharma, Jeffrey B. Jones

**Affiliations:** 1 Department of Plant Pathology, University of Florida, Gainesville, FL 32611, USA; poudel.mousami@ufl.edu (M.P.); smcduffee@ufl.edu (S.M.); gvmins@ufl.edu (G.V.M.); 2 Department of Horticultural Sciences, Gulf Coast Research and Education Center, Wimauma, FL 33598, USA

**Keywords:** recessive resistance, *Xanthomonas*, gene pyramiding, *bs5*, *bs6*, *bs8*, bacterial leaf spot

## Abstract

Bacterial spot of the pepper (BSP) and the tomato (BST) caused by multiple *Xanthomonas* spp. remains a major constraint to production of both crops worldwide. The widespread breakdown of dominant resistance genes, such as *Bs2*, due to the emergence of virulent races, like *Xanthomonas euvesicatoria* P6, has underscored the need for more durable, non-race-specific resistance. The recessive genes, *bs5*; *bs6*; and *bs8*, have emerged as promising alternatives, conferring broad-spectrum resistance without triggering a hypersensitive response. In this study, we systematically evaluated the individual and combinatorial effects of these three recessive resistance genes against three *Xanthomonas* species, *X. euvesicatoria* (*Xe*), *X. hortorum* pv. *gardneri* (*Xhg*), and *X. perforans* (*Xp*). Using near-isogenic lines (NILs) developed in the susceptible Early Calwonder (ECW) background, we assessed the in planta bacterial population growth and symptom development across a panel of eight genotypes carrying different gene combinations. Our results demonstrate that *bs5*, particularly when combined with either *bs6* or *bs8*, significantly reduces bacterial growth and disease severity across all three *Xanthomonas* species. The triple-stacked line (ECW568 (i.e., *bs5*, *bs6*, and *bs8*)) consistently displayed the strongest suppression of pathogen proliferation and symptom development. By contrast, *bs6* and *bs8*, alone or in combination, were largely ineffective. In some cases, combining *bs6* with *bs8* was less effective than *bs8* alone. These findings reinforce the central role of *bs5* in conferring quantitative resistance and highlight the additive benefit of pyramiding recessive resistance genes. Furthermore, we have demonstrated that these recessive resistance genes are effective in limiting the ability of the emerging pathogen, *X. perforans*, to grow *in planta*, and thus are predicted to offer a high level of resistance in the field. Our work provides key insights for breeding durable, broad-spectrum resistance into commercial pepper cultivars and offers a framework for integrated disease management strategies in the face of rapidly evolving bacterial pathogens

## 1. Introduction

Bacterial spot of the tomato (BST) and the pepper (BSP) caused by *Xanthomonas* spp. are very destructive diseases affecting pepper (*Capsicum annuum*) and tomato (*Solanum lycopersicum* L.) production worldwide [1,2]. BSP can lead to yield losses of up to 44% in peppers [3], whereas BST can cause yield losses close to 50% in tomatoes [4]. BSP and BST are caused by four bacterial species, *Xe*, *Xv*, *Xp*, and *Xhg* [2,5,6]. Historically, strains of *Xe*, *Xv*, and *Xhg* were considered pathogenic on both the pepper and the tomato, while *Xp* was thought to infect only the tomato [5]. However, the recent isolation of *Xp* strains from pepper fields in Alabama and Florida indicates a host range expansion for this species [6,7].

BSP manifests as brown, angular, water-soaked lesions on leaves, stems, and fruit, often resulting in defoliation and significant reductions in marketable fruit yield [8,9,10]. The disease is particularly severe in tropical and subtropical regions, where high temperatures and frequent rainfall favor its development [2,11]. The pathogen spreads via wind-driven rain, irrigation splash, and contaminated farm equipment, and it can persist in crop residues, weeds, and volunteer plants [12]. While copper-based bactericides have traditionally been used for BSP management, the emergence of copper-tolerant strains has reduced their efficacy. As a result, the development and deployment of resistant pepper varieties has become one of the most effective, environmentally sustainable, and economically viable strategies for controlling BSP [13].

Differential reactions in the pepper and tomato genotypes have delineated pathogenic races of *Xanthomonas*, with four races identified in the tomato and eleven races (P0 to P10) in the pepper [13]. To date, six monogenic dominant hypersensitive resistance genes (*BsT*, *Bs1*, *Bs2*, *Bs3*, *Bs4C*, *Bs7*) and three non-hypersensitive recessive resistance genes (*bs5*, *bs6*, *bs8*) have been identified in the pepper to combat bacterial leaf spot (BLS) [14]. Until the early 2000s, most commercial pepper cultivars relied on *Bs2* resistance to manage BLS. However, as bacterial populations evolved, the effectiveness of *Bs2*-mediated resistance declined, leading to its breakdown within a few years of widespread use [13,15]. Understanding the limitations of relying on a single resistance gene, breeding programs focused on pyramiding resistance genes in combinations, such as *Bs2* + *Bs1* and *Bs2* + *Bs3*, to improve durability [16]. However, the extensive use of these resistance genes exerted strong selective pressure on pathogen populations, leading to the adaptation of bacteria that either lacked the corresponding *avr* genes or carried mutated alleles to avoid recognition [15]. Despite these efforts, the emergence of race P6, which lacks *avrBs1*, *avrBs2*, and *avrBs3*, has rendered traditional dominant resistance genes in the pepper ineffective, making P6 virulent on all commercial pepper cultivars [17].

To address this challenge, researchers have identified three recessive resistance genes, *bs5*, *bs6*, and *bs8*, which provide alternative mechanisms of resistance against multiple *Xanthomonas* spp. [14,17]. The two recessive genes, *bs5* and *bs6*, confer broad-spectrum resistance against all races of *Xe*, including P6 and P10 [17], while *bs8* provides resistance against *Xhg* [14]. Unlike dominant resistance genes, which rely on the hypersensitive response (HR)-mediated recognition of bacterial effectors, *bs5*, *bs6*, and *bs8* suppress bacterial growth without triggering an HR, offering a durable, non-race-specific resistance mechanism [17,18]. Recent studies have fine-mapped these three recessive resistance genes in the pepper genome, providing essential insights for marker-assisted selection in breeding programs. The *bs5* gene was derived from *C. annum* PI 271322, and localized to a ~535 Kbp interval on chromosome 3; while *bs6*, originating from the C44 pepper line series (PI 264281 and PI 163192), was mapped to a ~666 Kbp interval on chromosome 6 [19]. Similarly, *bs8* was mapped to a 2.3-Mbp region on chromosome 11 [14].

Previous work has demonstrated the efficacy of *bs5* and *bs6* under specific conditions. For example, Vallejos et al. [18] showed that temperature has a significant impact on the effectiveness of these resistance genes. At 25 °C, both *bs5* and *bs6* restricted bacterial growth of *Xe* race P6 in near-isogenic lines ECW50R (*bs5*) and ECW60R (*bs6*), with *bs5* providing stronger resistance. However, at 30 °C, the ability of either gene alone to limit bacterial growth was significantly reduced, with *bs6* performing similarly to the susceptible control (ECW). Despite the negative effect of high temperature on single gene resistance, the combination of *bs5* and *bs6* demonstrated a synergistic effect, leading to enhanced resistance, even at 30 °C [18].

Recently, *Xp* has been isolated in pepper production fields, and is considered an emerging plant pathogen. Although copper bactericides are available, it will likely take a relatively short period of time before the strains acquire copper resistance, as has happened for the tomato pathogen [8]. Given it is genetically distinct from *Xe* [20], it is difficult to extrapolate how the available resistance in the pepper will behave when exposed to the new *Xp* strains on the pepper. Therefore, it is necessary to determine how *Xp* responds to the pepper genotypes containing *bs5*, *bs6*, and *bs8*.

In this study, we build on these insights by pyramiding *bs5*, *bs6*, and/ or *bs8* in the pepper and evaluate their individual and combined contributions to bacterial spot resistance. We challenged a set of NILs, each containing different combinations of these recessive genes, with three *Xanthomonas* species (*Xe*, *Xp* and *Xhg*). Our objectives were to determine (i) the extent of resistance conferred by each gene alone, (ii) whether combining these genes yields additive or synergistic resistance effects, and (iii) the consistency of these effects across different *Xanthomonas* species. By conducting replicated experiments and comparing the results across two independent trials, we also aimed to assess the reproducibility of the resistance phenotypes. The findings are discussed in the context of breeding strategies for durable bacterial spot resistance in the pepper.

## 2. Materials and Methodology

### 2.1. Plant Genetic Materials

Near isogenic lines in the Early Calwonder (ECW) background carrying recessive resistance genes *bs5*, *bs6*, and *bs8* were developed through marker-assisted selection. ECW is a bacterial spot-susceptible bell pepper cultivar lacking resistance genes. ECW50R (carrying *bs5* introgressed from PI 271322) [18], ECW60R (*bs6* introgressed from *C. chinense* C-44) [19], and ECW80R (*bs8* introgressed from PI 163192) [14] were intercrossed to combine loci. First, ECW50R was crossed with ECW60R to obtain F_1_ progeny heterozygous for *bs5* and *bs6*. This F_1_ was then crossed with ECW80R to introduce *bs8*, generating progeny heterozygous for all three loci. Self-pollination of these F_1_ plants produced a segregating F_2_ population, from which individuals homozygous for all three resistance alleles (*bs5*, *bs6*, *bs8*) were identified via molecular markers (Table 1). After additional selfing and selection, a triple homozygous NIL designated ECW568R (*bs5*, *bs6*, *bs8*) was established.

To create a full panel of lines with different gene combinations, ECW568R was backcrossed to the wild-type ECW, and the resulting F1 (heterozygous for *bs5*, *bs6*, *bs8*) was selfed to produce a large F_2_ population. Marker screening of the F_2_ population identified plants homozygous for each possible combination of the three genes. Eight genotypic classes were selected as follows: (1) ECW5 (*bs5*), (2) ECW6 (*bs6*), (3) ECW8 (*bs8*), (4) ECW56 (*bs5*, *bs6*), (5) ECW58 (*bs5*, *bs8*), (6) ECW68 (*bs6*, *bs8*), (7) ECW568 *(bs5*, *bs6*, *bs8*), and (8) ECW (susceptible control).

### 2.2. Bacterial Strains and Inoculation

These eight genotypes were then inoculated, separately, with three different *Xanthomonas* species, Xe157, *Xg444*, and Xp706. Bacterial cultures were grown on nutrient agar (NA) (Difco^TM^, Becton Dickinson & Co, Sparks, MD, USA) at 28 °C for 24 h. Cells from fresh cultures were suspended in sterile tap water and adjusted to 10^8^ CFU/mL by measuring the optical density at OD_600_ = 0.3 using a Spectronic 20 Genesys spectrophotometer (Spectronic Instruments, Rochester, NY, USA). These bacterial suspensions were further diluted in sterile tap water to a final concentration of 10^5^ CFU/mL. Pepper plants (6–8 weeks old) were grown in a climate-controlled greenhouse or an ambient greenhouse. For each pepper genotype × bacterial strain combination, fully expanded young leaves were infiltrated on the abaxial side using a syringe and hypodermic needle with the appropriate bacterial suspension. Each leaf was infiltrated with a bacterial suspension to allow for sampling a small, infiltrated area (~1 cm^2^). Most experiments were conducted under a high temperature regimen (28–35 °C) to simulate typical warm field conditions conducive to bacterial spot development. However, for *Xhg* (*Xg444*), a second set of experiments was also performed in a greenhouse at a lower temperature (18–28 °C), given that *Xhg* is found in cooler environments [14].

### 2.3. Experimental Design and Data Collection

The experiment included eight pepper genotypes, each evaluated in most experiments at four time points 0, 3, 6, and 9 days post-inoculation (dpi), resulting in 32 individual plants per trial. For each genotype-day combination, one plant was used, and three leaves were sampled per plant to serve as replicates for bacterial quantification. Leaf samples (1 cm^2^ disks from the infiltrated area) were harvested, and the infiltrated areas were macerated in 1 mL sterile tap water. Serial 10-fold dilutions were plated on NA, and after 3 days of incubation at 28 °C, bacterial colonies were counted to determine population size (CFU/cm^2^ leaf area). Colony counts were log_10_-transformed for statistical analysis. Symptom development was visually monitored, and representative leaves were photographed between 7–9 dpi to document disease severity (chlorosis/necrosis).

### 2.4. Statistical Analysis

Data from the two independent experiments were analyzed separately to evaluate the consistency of genotype effects across replicates. Since the relative performance of genotypes was consistent between trials, the results are presented separately in the Figures below to allow for a visual comparison, while the standard error bars in each graph represent variations among the biological replicates within each experiment. For each pathogen, the area under the population progress curve (AUPPC) was calculated for each genotype using the trapezoidal method [21] across 0, 3, 6, and 9 dpi. The resulting AUPPC values were subjected to one-way analysis of variance (ANOVA), followed by Student–Newman–Keuls (SNK) post hoc tests to determine the statistically significant differences among genotypes. All statistical analyses were conducted using the Agricole package of R studio V 4.1.0, and letter groupings (a, b, c, etc.) indicate statistically homogeneous subsets of genotypes at α = 0.05. Unless otherwise noted, data are reported as mean ± standard error, based on three biological replicates per genotype and time point within each experiment. The two experiments were conducted independently under similar conditions and produced reproducible results, confirming the robustness of the genotype performance patterns.

An unstructured correlation structure was also applied to the residuals to account for the repeated measures of leaves coming from the same genotype and for variances across time. If significant interactions were found, a slice test and Tukey’s pairwise comparison procedure were used on the data to further test the significance of interactions (Supplementary Figure and Table S5–S19).

## 3. Results

### 3.1. Effect of Recessive Resistance Gene Combinations on Bacterial Multiplication and Disease Development Caused by X. euvesicatoria

**Figure 1:**
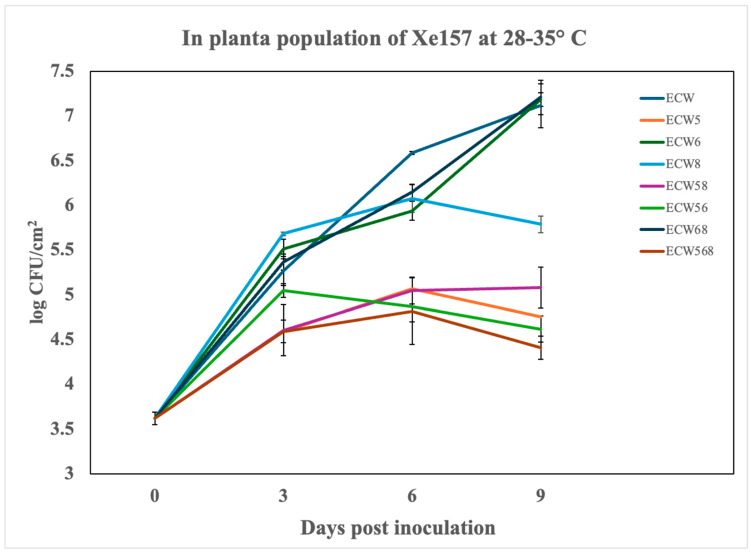
*In planta* growth of the *Xanthomonas euvesicatoria* strain Xe157 infiltrated at 10^5^ CFU/mL in the leaves of susceptible and resistant pepper lines and incubated under greenhouse conditions ranging from 28–35 °C. Genotypes include ECW, Early Calwonder, ECW5 (*bs5*), ECW6 (*bs6*), ECW8 (*bs8*), ECW58 (*bs5* + *bs8*), ECW56 (*bs5* + *bs6*), ECW68 (*bs6* + *bs8*), and ECW568 (*bs5* + *bs6* + *bs8*). Each point represents the mean of three biological replicates (individual plants), and error bars indicate standard error (SE) across these replicates. Results from Experiment 2 demonstrating reproducibility are provided in Supplementary Figure S1.

Under high-temperature conditions (28–35 °C) conducive to aggressive infection by *Xe*, significant reductions in bacterial growth were observed in the pepper genotypes carrying *bs5*. By 6 dpi, ECW5 (*bs5* alone) showed a ~33-fold reduction in the in planta *Xe* population compared to the susceptible ECW control (Figure 1). Stacking *bs5* with *bs6* or *bs8* further enhanced resistance, with ECW56 (*bs5* + *bs6*) and ECW58 (*bs5* + *bs8*) suppressing bacterial growth by ~52-fold and ~35-fold, respectively. The triple-stack ECW568 (*bs5* + *bs6* + *bs8*) demonstrated the highest resistance, achieving around a ~60-fold reduction in bacterial titers at 6 dpi. By contrast, genotypes lacking *bs5*, such as ECW6 (*bs6* alone) and ECW68 (*bs6* + *bs8*), supported high bacterial populations statistically indistinguishable from the susceptible control, highlighting the critical role of *bs5* in restricting *Xe* growth. A quantitative analysis using the AUPPC confirmed these observations. ECW568 had the lowest AUPPC values, followed closely by ECW56 and ECW58, with significant statistical separation between genotypes based on a Student–Newman–Keuls (SNK) analysis (Table 2). A second independent experiment, which further confirms these observations and demonstrates reproducibility, is provided in Supplementary Figure S1 and Supplementary Table S1.

**Figure 2:**
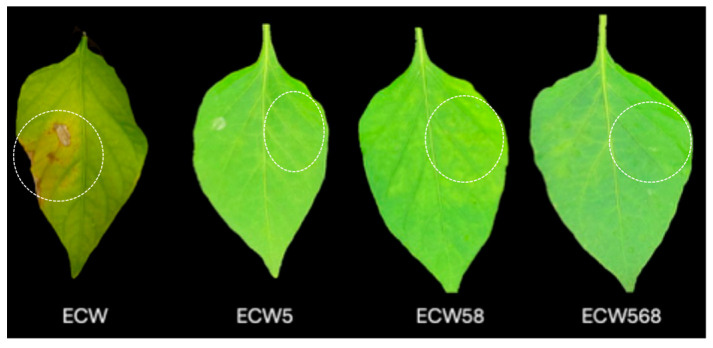
Symptom development of *Xanthomonas euvesicatoria* on the pepper genotypes at 7 days post-inoculation. White dashed circles represent the inoculated areas on the leaves. Representative leaves from the susceptible ECW, Early Calwonder line and near-isogenic lines ECW5 (*bs5*), ECW58 (*bs5* + *bs8*), and ECW568 (*bs5* + *bs6* + *bs8*) were infiltrated with the *X. euvesicatoria* strain Xe157 and incubated at 28–35 °C. All three resistance lines exhibited reduced symptom development compared to ECW, with ECW568 showing the least chlorosis and necrosis.

Visual disease symptoms mirrored the bacterial count data. As shown in Figure 2, ECW plants exhibited prominent chlorosis and lesion development at 7 days post-inoculation. By comparison, ECW5 (*bs5*) and ECW58 (*bs5* + *bs8*) showed moderate symptom reduction. Notably, ECW568 (*bs5* + *bs6* + *bs8*) demonstrated near-complete suppression of visible symptoms, remaining green and healthy in appearance. These qualitative symptom observations aligned closely with the quantitative bacterial counts, emphasizing that, although *bs5* alone confers strong resistance, stacking it with *bs6* or *bs8* significantly enhances overall disease suppression. The consistent and reproducible performance of ECW568 across independent trials strongly validates the effectiveness of this gene pyramid.

### 3.2. Effect of Recessive Resistance Gene Combinations on Multiplication and Disease Development Caused by X. hortorum pv. gardneri

**Figure 3:**
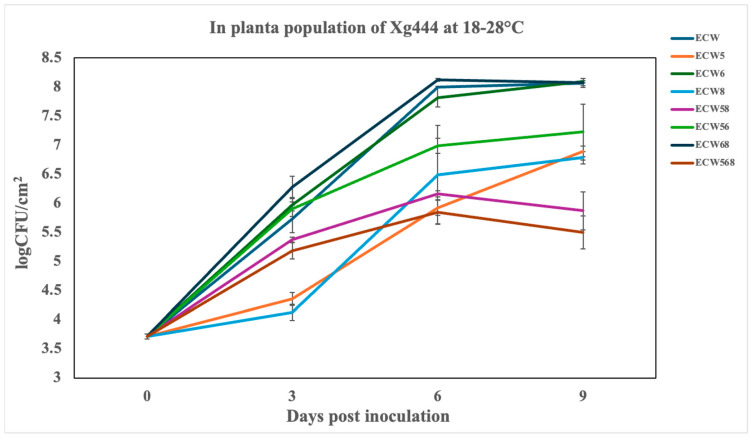
*In planta* growth of the *Xanthomonas hortorum* pv. *gardneri* strain *Xg444* infiltrated at 10^5^ CFU/mL in the leaves of susceptible and resistant pepper lines, and incubated under greenhouse conditions ranging from 18–28 °C. Genotypes include ECW, Early Calwonder, ECW5 (*bs5*), ECW6 (*bs6*), ECW8 (*bs8*), ECW58 (*bs5* + *bs8*), ECW56 (*bs5* + *bs6*), ECW68 (*bs6* + *bs8*), and ECW568 (*bs5* + *bs6* + *bs8*). Each point represents the mean of three biological replicates (individual plants), and error bars indicate standard error (SE) across these replicates. Results from Experiment 2 demonstrating reproducibility are provided in Supplementary Figure S2.

Under cooler incubation conditions (18–28 °C), all pepper genotypes carrying *bs5*, *bs8*, or their combinations exhibited substantial reductions in bacterial populations of the *Xhg* strain *Xg444* relative to the susceptible control ECW at 6 dpi. ECW5 (*bs5*) and ECW8 (*bs8*) individually reduced bacterial growth by approximately 117-fold and 32-fold, respectively. Combining these genes showed moderate resistance, as ECW58 (*bs5* + *bs8*) showed an approximately 10-fold reduction (Figure 3). The triple-stacked genotype ECW568 (*bs5* + *bs6* + *bs8*) displayed the greatest resistance, with a ~140-fold suppression in bacterial multiplication. By comparison, ECW56 (*bs5* + *bs6*) showed significant suppression at ~68-fold, indicating an additive or synergistic interaction. By contrast, genotypes carrying *bs6* alone (ECW6) or in combination with *bs8* (ECW68) showed limited to no suppression of *Xhg*; notably, ECW68 even exhibited a slight increase in bacterial populations relative to ECW, suggesting a negative interaction when combining *bs6* and *bs8* in the absence of *bs5*.

The AUPPC values reinforced these trends, with ECW568 and ECW56 having the lowest values (Table 3); a statistical analysis using SNK tests confirmed significant differences among these genotypes (α = 0.05). A second independent experiment, which further confirms these observations and demonstrates reproducibility, is provided in Supplementary Figure S2 and Supplementary Table S2. Overall, these results demonstrate that *bs5* and *bs8* are effective individually, but their combination offers additive or synergistic protection. By contrast, *bs6* appears to have minimal or inconsistent effects under low-temperature conditions. The high reproducibility across experiments reinforces the robustness of *bs5-* and *bs8*-mediated resistance to *Xhg* in cooler environments.

**Figure 4:**
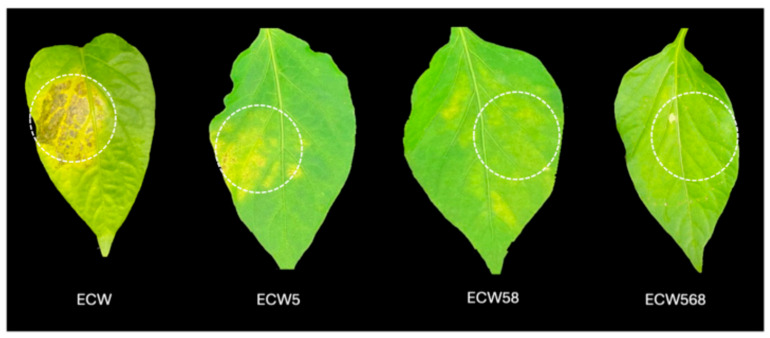
Visual symptom development of *Xanthomonas hortorum* pv. *gardneri* on the pepper genotypes at 7 days post-inoculation. White dashed circles represent the inoculated areas on the leaves. Representative leaves from susceptible ECW and near-isogenic lines ECW5 (*bs5*), ECW58 (*bs5* + *bs8*), and ECW568 (*bs5* + *bs6* + *bs8*) were infiltrated with the *X. hortorum* pv. *gardneri* strain *Xg444* and incubated in greenhouse at 20–25 °C. ECW displayed severe chlorosis and necrosis, while ECW5 showed moderate disease. By contrast, ECW58 and ECW568 showed markedly reduced symptom expression, consistent with their lower bacterial titers and supporting the additive contribution of *bs5* and *bs8* to effective disease suppression.

These findings were corroborated by symptom observations on inoculated leaves. By 7 dpi, ECW plants exhibited characteristic water-soaked spots, coalescing into large chlorotic and necrotic patches (Figure 4). ECW5 (*bs5*) leaves showed only moderate disease with fewer and smaller lesions than ECW. ECW58 (*bs5* + *bs8*) and ECW568 (*bs5* + *bs6* + *bs8*) had dramatically reduced symptoms, showing limited to faint chlorotic lesions. These visual results reinforce the quantitative data, highlighting the additive contribution of *bs5* and *bs8* in suppressing both bacterial growth and symptom expression.

However, in an experiment where plants were challenged with *Xg444* under higher temperature conditions (25–35 °C), overall disease severity increased, and even the resistant lines showed elevated bacterial growth. Under those conditions, only the triple combination ECW568 maintained substantially lower *Xhg* populations compared to ECW and other combinations. Genotypes carrying *bs5* alone or in combination with *bs8* showed partial suppression, whereas genotypes lacking *bs5*, including ECW6, ECW8, and ECW68 were essentially ineffective (Supplementary Figure S3). Across both temperature scenarios, ECW568 consistently maintained the lowest in planta bacterial populations.

### 3.3. Effect of Recessive Resistance Gene Combinations on Multiplication and Disease Development Caused by X. perforans

**Figure 5:**
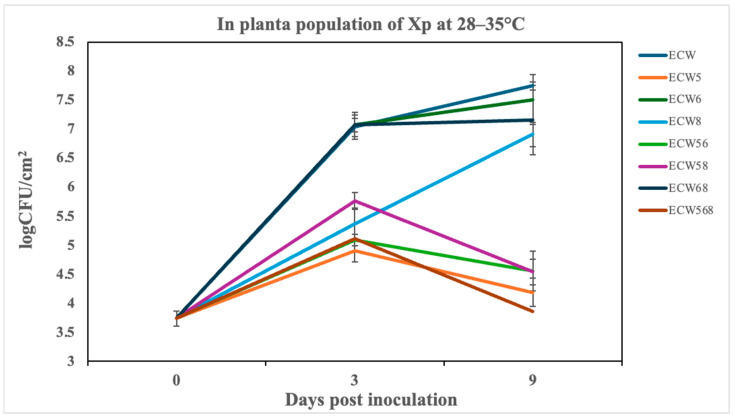
*In planta* bacterial populations of the *Xanthomonas perforans* strain Xp706 infiltrated at 10^5^ CFU/mL in leaves of the susceptible and resistant pepper genotypes at 0, 3, and 9 days post-inoculation under greenhouse conditions ranging from 28–35 °C incubation. Genotypes include ECW (susceptible control), ECW5 (*bs5*), ECW6 (*bs6*), ECW8 (*bs8*), ECW56 (*bs5* + *bs6*), ECW58 (*bs5* + *bs8*), ECW68 (*bs6* + *bs8*), and ECW568 (*bs5* + *bs6* + *bs8*). Each point represents the mean of three biological replicates (individual plants), and error bars indicate standard error (SE) across replicates. Results from Experiment 2 demonstrating reproducibility are provided in Supplementary Figure S4.

Under high-temperature conditions (28–35 °C), the pepper genotypes carrying *bs5*, alone or stacked with *bs6* and/or *bs8*, exhibited significantly lower *Xp* populations compared to the susceptible control ECW (Figure 5). At 9 dpi, ECW5 (*bs5* alone) displayed approximately a 3600-fold reduction. Resistance was enhanced in dual-gene combinations, with ECW58 (*bs5* + *bs8*) and ECW56 (*bs5* + *bs6*) reducing bacterial populations by about 1555-fold and 1606-fold, respectively. The triple-stacked genotype ECW568 (*bs5* + *bs6* + *bs8*) exhibited robust bacterial suppression, showing around a 7771-fold reduction relative to ECW. Genotypes lacking *bs5*, including ECW6 (*bs6*), ECW8 (*bs8*), and ECW68 (*bs6* + *bs8*), provided negligible bacterial suppression.

**Figure 6:**
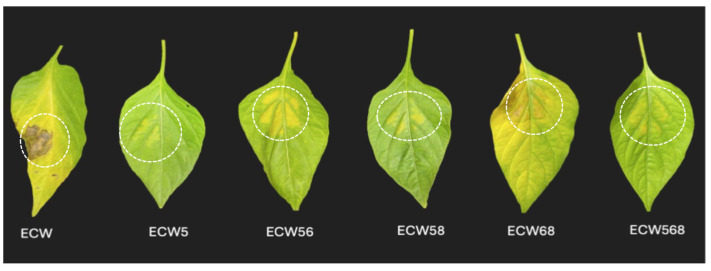
Visual symptom development caused by the *Xanthomonas perforans* strain Xp706 on the pepper genotypes at 9 days post-inoculation under greenhouse conditions ranging from 28–35 °C. White dashed circles represent the inoculated areas on the leaves. Representative leaves from the susceptible cultivar ECW and near-isogenic lines ECW5 (*bs5*), ECW56 (*bs5* + *bs6*), ECW58 (*bs5* + *bs8*), ECW568 (*bs5* + *bs6* + *bs8*), and ECW68 (*bs6* + *bs8*) were infiltrated with Xp706. ECW exhibited severe chlorosis and necrosis, while lines carrying *bs5*, alone or in combination, displayed visibly reduced symptoms. By contrast, ECW68 developed symptoms similar to ECW, reinforcing that *bs6* and *bs8* together are insufficient for effective resistance.

These quantitative findings were confirmed by cumulative bacterial growth measurements (AUPPC; Table 4), with ECW5 and ECW568 consistently showing the lowest cumulative bacterial populations, followed closely by ECW56 and ECW58. Whereas ECW6, ECW8, and ECW68 showed high AUPPC values similar to the susceptible ECW. A statistical analysis using SNK multiple comparisons confirmed significant differences among the genotypes (α = 0.05). A second independent experiment, which further confirms these observations and demonstrates reproducibility, is provided in Supplementary Figure S4 and Supplementary Table S4. Visual symptom assessments at 9 dpi mirrored these quantitative values (Figure 6). ECW leaves developed severe chlorosis and necrosis, while *bs5*-containing lines displayed visibly reduced symptoms. ECW5 showed moderate disease, whereas ECW56, ECW58, and particularly ECW568, had minimal lesion development. By contrast, ECW68 was indistinguishable from ECW, reinforcing that stacking *bs6* and *bs8* without *bs5* is ineffective.

## 4. Discussion

This study demonstrates the effectiveness of pyramiding recessive resistance genes (*bs5*, *bs6*, and *bs8*) to combat bacterial spot disease in the pepper caused by multiple *Xanthomonas* species. Furthermore, we provide information for the first time on the use of these resistance genes for control of the emerging pepper pathogen, *X. perforans.* Across two independent experiments with three *Xanthomonas* spp., we found that *bs5* was the critical player in recessive resistance under both moderate and elevated temperature regimes. When combined with *bs6* or *bs8*, *bs5* consistently conferred enhanced protection, substantially reducing bacterial populations and minimizing visible disease symptoms. The triple-stacked genotype (ECW568), carrying *bs5*, *bs6*, and *bs8*, exhibited the most robust and consistent suppression of the in planta bacterial multiplication for all three *Xanthomonas* pathogens, regardless of environmental conditions. These findings underscore the additive or synergistic benefits of gene pyramiding and highlight the utility of recessive resistance alleles in developing durable, broad-spectrum bacterial spot resistance in the pepper.

Although the presence of *bs5* was consistently associated with significant reductions in the in planta bacterial multiplication across all three *Xanthomonas* species (*Xe*, *Xhg*, and *Xp*), particularly under high-temperature conditions, *bs6* and *bs8* alone provided limited or inconsistent protection. These results align with the findings of Vallejos et al. [18], who demonstrated that, while *bs5* alone provided strong partial resistance at moderate temperatures (~25 °C), its effectiveness was significantly compromised at elevated temperatures (30 °C), and that only the combined presence of *bs5* and *bs6* conferred near-complete resistance under heat stress, indicative of positive epistasis at high temperature. Our findings similarly reinforce their conclusion that gene stacking, especially combining *bs5* with other recessive genes, is essential for maintaining stable, effective resistance under challenging environmental conditions.

Recent transcriptomic analyses provide mechanistic insights supporting the broad-spectrum and temperature-resilient nature of *bs5*-mediated resistance [22]. Specifically, *bs5* was found to rapidly activate a robust basal defense network characterized by the upregulation of key genes involved in pathogen-associated molecular pattern-triggered immunity (PTI) [23]. Among these were the pattern recognition receptor FLS2, calcium-dependent signaling components (CDPKs, CaM/CML), NADPH oxidase (RbohD), MAP kinase cascades (MPK3, MPK4), and defense-associated transcription factors (WRKY29, WRKY33, and WRKY22). These genes collectively promote a rapid oxidative burst and an enhanced expression of defense-related genes, such as PR-1, without relying on a classical hypersensitive response. This PTI-centered defense signature, driven by *bs5*, helps in explaining the stable and non-race-specific nature of resistance observed across the various *Xanthomonas* spp. Furthermore, the activation of stress-responsive pathways, including jasmonate and ethylene signaling early in infection, along with the modulation of salicylic acid signaling, supports the hypothesis that *bs5* resistance remains effective under thermal stress conditions. This mechanistic understanding aligns closely with our observed field phenotypes, providing a robust molecular basis for breeding strategies centered around *bs5*.

Interestingly, the combination of *bs6* and *bs8* (ECW68) not only failed to improve resistance but in some cases appeared to enhance bacterial growth, particularly against *Xhg* at lower temperatures. This suggests a possible antagonistic or epistatic interaction between *bs6* and *bs8* in the absence of *bs5*. Such interactions highlight the importance of empirical validation when combining resistance loci, as not all combinations yield additive effects. Breeders should be cautious when deploying gene stacks and consider the functional compatibility of resistance alleles, especially those whose mechanisms are not yet fully characterized.

In contrast to ECW68, the triple-stacked genotype ECW568 showed an approximately 2.5 log-fold reduction in bacterial populations compared to the susceptible control ECW against *Xhg*, closely matching the performance reported by [14] in their study with genotype PI163192, which also carries the recessive resistance genes *bs5*, *bs6*, and *bs8*. This similarity strongly supports the reproducibility and reliability of resistance mediated by these recessive gene combinations.

The effectiveness of *bs5*-based combinations against *Xp* is particularly noteworthy. Once considered a tomato-specific pathogen, *Xp* has expanded its host range to the pepper in recent years [6,7]. The aggressive nature of this species, coupled with its increasing prevalence in warm production regions, poses a serious challenge to disease management. In our study, the triple-gene combination (ECW568) reduced *Xp* populations by nearly eight thousand fold in one experiment at day 9 (Figure 4) and consistently outperformed all other genotypes. This result reinforces the need for forward-looking breeding strategies that anticipate pathogen evolution and emerging threats.

Our use of replicated, independently conducted experiments strengthens the reliability of these findings. While quantitative reductions in pathogen load varied somewhat between experiments, particularly for *Xp*, the relative performance of genotypes remained stable. This consistency supports the robustness of *bs5*-mediated resistance and validates the use of the AUPPC and the log-transformed bacterial counts as quantitative disease metrics in pepper–*Xanthomonas* interaction studies.

Overall, this study provides strong evidence for the strategic use of *bs5*, particularly when combined with *bs6* and/or *bs8*, in pepper breeding programs. The deployment of multiple recessive resistance genes, rather than a reliance on dominant, HR-based single genes, offers a more sustainable approach to managing bacterial spot. Recessive genes are less likely to exert strong selection pressure on pathogen populations, and gene pyramiding can buffer against partial breakdowns of individual alleles. As climate change accelerates and pathogen populations become increasingly diverse and aggressive, stacking multiple quantitative resistance loci may prove to be one of the most reliable defenses available to breeders and growers. Future work should explore the molecular basis of observed interactions between resistance genes, assess the long-term stability of these combinations under field conditions, and integrate marker-assisted selection for the efficient deployment of *bs5*-centered gene stacks. By leveraging these insights, breeders can accelerate the development of durable, broad-spectrum bacterial spot resistance in commercial pepper cultivars.

## 5. Conclusions

This study demonstrates that pyramiding recessive resistance genes, particularly *bs5* with *bs6* and *bs8*, provides strong, additive suppression of BSP caused by diverse *Xanthomonas* species. While *bs5* alone significantly reduces bacterial growth and symptom development, combining it with additional resistance loci enhances overall protection and broadens effectiveness across pathogens. The triple-stacked line (*bs5* + *bs6* + *bs8*) consistently exhibited the strongest resistance, including under elevated temperature conditions where single- and two-gene combinations often failed. These findings reinforce the importance of gene stacking to stabilize resistance expression, especially in the face of environmental variability and evolving pathogen populations. Ultimately, *bs5*-centered pyramiding offers a promising framework for breeding pepper cultivars with more durable, broad-spectrum resistance. Further integration with marker-assisted selection and field-level evaluation will be key to translating this strategy into sustainable disease management solutions for growers.

## Figures and Tables

**Table 1:** Molecular markers used in this study.

Name		Sequence	Position (CM334)	Position (UCD10x)	Polymorphism	Reference
*bs5*	JW1_F1	ACCGAGTCTCATGCTGTTTC	~760,000	~390,000	CCAAGAG>C delCAAGAG	This study
	JW1_R1	CAACACTTTGCGTACAGATCATT	~760,000	~390,000	CCAAGAG>C delCAAGAG	
*bs6*	190.99_F1/6g_H178.55	TTCTCATTATCCGTATCATTACCC	190,990,000	178,550,000	AA>GG	Sharma et al. [19]
	190.99_R1/6g_H178.55	CGTTCCACAAACGACATCT	190,990,000	178,550,000	AA>GG	
*bs8*	bsh_260.54_F1	GGGGTACTGAGAGTGACCCTA	260,544,417	232,640,000	A>G	This study
	bsh_260.54_R1	CAAAGCCACGTGGTTCCG	260,544,417	232,640,000	A>G	

**Table 2:** Area under the population progress curve (AUPPC) for the *Xanthomonas euvesicatoria* strain Xe157 in the pepper genotypes incubated under greenhouse conditions ranging from 28 to 35 °C. The AUPPC was calculated from 0 to 9 days post-inoculation using the trapezoidal method. Genotypes include ECW (susceptible control), ECW5 (*bs5*), ECW6 (*bs6*), ECW8 (*bs8*), ECW56 (*bs5* + *bs6*), ECW58 (*bs5* + *bs8*), ECW68 (*bs6* + *bs8*), and ECW568 (*bs5* + *bs6* + *bs8*). Different letters indicate statistically significant differences among genotypes based on Student–Newman–Keuls (SNK) multiple comparisons at α = 0.05. Supplementary Table S1 provides the AUPPC data from Experiment 2 to illustrate the reproducibility of the results.

Genotype	AUPPC	SNK Group
ECW568	40.26	c
ECW5	41.53	c
ECW56	42.00	c
ECW58	42.10	c
ECW8	49.11	b
ECW6	50.55	ab
ECW68	50.78	ab
ECW	51.66	a

**Table 3:** Area under the population progress curve (AUPPC) for the *Xanthomonas hortorum* pv. *gardneri* strain *Xg444* in the pepper genotypes incubated under greenhouse conditions ranging from 18 to 28 °C. The AUPPC was calculated from 0 to 9 days post-inoculation using the trapezoidal method. Genotypes include ECW (susceptible control), ECW5 (*bs5*), ECW6 (*bs6*), ECW8 (*bs8*), ECW56 (*bs5* + *bs6*), ECW58 (*bs5* + *bs8*), ECW68 (*bs6* + *bs8*), and ECW568 (*bs5* + *bs6* + *bs8*). Different letters indicate statistically significant differences among genotypes based on Student–Newman–Keuls (SNK) multiple comparisons at α = 0.05. Supplementary Table S2 provides the AUPPC data from Experiment 2 to illustrate the reproducibility of the results.

Genotype	AUPPC	SNK Group
ECW568	46.95	c
ECW5	47.46	c
ECW56	49.00	c
ECW8	50.80	c
ECW58	55.09	b
ECW	56.08	b
ECW6	59.10	ab
ECW68	60.9	a

**Table 4:** Area under the population progress curve (AUPPC) for the *Xanthomonas perforans* strain Xp706 in the pepper genotypes under 28–35 °C incubation. The AUPPC was calculated from 0 to 9 days post-inoculation using the trapezoidal method. Genotypes include ECW (susceptible control), ECW5 (*bs5*), ECW6 (*bs6*), ECW8 (*bs8*), ECW56 (*bs5* + *bs6*), ECW58 (*bs5* + *bs8*), ECW68 (*bs6* + *bs8*), and ECW568 (*bs5* + *bs6* + *bs8*). Different letters indicate statistically significant differences among genotypes based on Student–Newman–Keuls (SNK) multiple comparisons at α = 0.05. Supplementary Table S4 provides the AUPPC data from Experiment 2 to illustrate the reproducibility of results.

Genotype	AUPPC	SNK Group
ECW568	40.13	d
ECW5	40.22	d
ECW56	42.14	d
ECW58	45.13	c
ECW8	50.45	b
ECW68	58.87	a
ECW6	59.95	a
ECW	60.47	a

## Data Availability

The original contributions presented in this study are included in the article. Further inquiries can be directed to the corresponding authors.

## Supplementary Materials

The following supporting information can be downloaded at: https://www.mdpi.com/article/10.3390/plants14162559/s1, Figure S1. *In planta* growth of *Xanthomonas euvesicatoria* (strain Xe157, 10^5^ CFU/ml) infiltrated in leaves of susceptible and resistant pepper lines over 9 dpi, incubated in greenhouse at 28–35 °C (Experiment 2). Genotypes include ECW, Early CalWonder; ECW5 (*bs5*); ECW6 (*bs6*), ECW8 (*bs8*), ECW58 (*bs5* + *bs8*), ECW56 (*bs5* + *bs6*), ECW68 (*bs6* + *bs8*), ECW568 (*bs5* + *bs6* + *bs8*). Each point represents the mean of three biological replicates (individual plants), and error bars indicate standard error (SE) across these replicates. Table S1: Area under the pathogen progress curve (AUPPC) for *Xanthomonas euvesicatoria* strain Xe157 in pepper genotypes, incubated in greenhouse at 28–35 °C (Experiment 2). AUPPC was calculated from 0 to 9 days post-inoculation using the trapezoidal method. Genotypes include ECW (susceptible control), ECW5 (*bs5*), ECW6 (*bs6*), ECW8 (*bs8*), ECW56 (*bs5* + *bs6*), ECW58 (*bs5* + *bs8*), ECW68 (*bs6* + *bs8*), and ECW568 (*bs5* + *bs6* + *bs8*). Different letters indicate statistically significant differences among genotypes based on Student–Newman–Keuls (SNK) multiple comparisons at α = 0.05. Figure S2. *In planta* growth of *Xanthomonas hortorum* pv. *gardneri* (strain *Xg444*, 10^5^ CFU/mL) infiltrated in leaves of susceptible and resistant pepper lines and incubated under greenhouse conditions ranging from 18–28 °C for 0,3,6 and 9 days (Experiment 2). Genotypes include ECW, Early CalWonder; ECW5 (*bs5*); ECW6 (*bs6*), ECW8 (*bs8*), ECW58 (*bs5* + *bs8*), ECW56 (*bs5* + *bs6*), ECW68 (*bs6* + *bs8*), ECW568 (*bs5* + *bs6* + *bs8*). Each point represents the mean of three biological replicates (individual plants), and error bars indicate standard error (SE) across these replicates. Table S2: Area under the pathogen progress curve (AUPPC) for *Xanthomonas hortorum* pv. gardneri strain *Xg444* in pepper genotypes under 18–28 °C incubation (Experiment 2). AUPPC was calculated from 0 to 9 days post-inoculation using the trapezoidal method. Genotypes include ECW, Early CalWonder; ECW5 (*bs5*); ECW6 (*bs6*), ECW8 (*bs8*), ECW58 (*bs5* + *bs8*), ECW56 (*bs5* + *bs6*), ECW68 (*bs6* + *bs8*), ECW568 (*bs5* + *bs6* + *bs8*). Different letters indicate statistically significant differences among genotypes based on Student–Newman–Keuls (SNK) multiple comparisons at α = 0.05. Figure S3. *In planta* growth of *Xanthomonas hortorum* pv. *gardneri* (strain *Xg444*, 10^5^ CFU/mL) infiltrated in leaves of susceptible and resistant pepper lines and incubated under greenhouse conditions ranging from 28–35 °C for 0, 3, 6 and 9 days. Genotypes include ECW, Early CalWonder; ECW5 (*bs5*); ECW6 (*bs6*), ECW8 (*bs8*), ECW58 (*bs5* + *bs8*), ECW56 (*bs5* + *bs6*), ECW68 (*bs6* + *bs8*), ECW568 (*bs5* + *bs6* + *bs8*). Each point represents the mean of three biological replicates (individual plants), and error bars indicate standard error (SE) across these replicates. Table S3: Area under the pathogen progress curve (AUPPC) for *Xanthomonas hortorum* pv. *gardneri* strain *Xg444* in pepper genotypes incubated under greenhouse conditions ranging from 28–35 °C (Experiment 2). AUPPC was calculated from 0 to 9 days post-inoculation using the trapezoidal method. Genotypes include ECW, Early CalWonder; ECW5 (*bs5*); ECW6 (*bs6*), ECW8 (*bs8*), ECW58 (*bs5* + *bs8*), ECW56 (*bs5* + *bs6*), ECW68 (*bs6* + *bs8*), ECW568 (*bs5* + *bs6* + *bs8*). Different letters indicate statistically significant differences among genotypes based on Student–Newman–Keuls (SNK) multiple comparisons at α = 0.05. Figure S4. *In planta* bacterial populations of *Xanthomonas perforans* strain Xp706 infiltrated with 10^5^ CFU/mL in leaves of susceptible and resistant pepper genotypes at 0, 3, and 7 days incubated under greenhouse conditions ranging from 28–35 °C (Experiment 2). Genotypes include ECW, Early CalWonder; ECW5 (*bs5*); ECW6 (*bs6*), ECW8 (*bs8*), ECW58 (*bs5* + *bs8*), ECW56 (*bs5* + *bs6*), ECW68 (*bs6* + *bs8*), ECW568 (*bs5* + *bs6* + *bs8*). Each point represents the mean of three biological replicates (individual plants), and error bars indicate standard error (SE) across replicates. Table S4. Area under the pathogen progress curve (AUPPC) for *Xanthomonas perforans* strain Xp706 in pepper genotypes incubated at 28–35 °C (Experiment 2). AUPPC was calculated from 0 to 7 days post-inoculation using the trapezoidal method. Genotypes include ECW, Early CalWonder; ECW5 (*bs5*); ECW6 (*bs6*), ECW8 (*bs8*), ECW58 (*bs5* + *bs8*), ECW56 (*bs5* + *bs6*), ECW68 (*bs6* + *bs8*), ECW568 (*bs5* + *bs6* + *bs8*). Different letters indicate statistically significant differences among genotypes based on Student–Newman–Keuls (SNK) multiple comparisons at α = 0.05. Table S5. Type III tests of fixed effects for *Xanthomonas euvesicatoria* (Xe157) population across pepper genotypes and time. All fixed effects—day, genotype, and their interaction—were highly significant (*p* < 0.0001), indicating strong temporal and genotypic differences in bacterial growth. Supplemental Table S6. Slice test of least squares means for *X. euvesicatoria* at 3 dpi. Significant differences among genotypes were detected at 3 days post-inoculation (*p* < 0.0001). Figure S5. Tukey grouping of least square means for bacterial populations in pepper genotypes inoculated with *Xanthomonas euvesicatoria* (Xe157) at 3 days post-inoculation.LS-means estimates (log CFU/cm^2^) are grouped using Tukey’s HSD test (α = 0.05). Bars sharing the same color indicate no significant difference in bacterial population levels. Genotypes D (ECW8), G (ECW68), A (ECW), and C(ECW6) exhibited significantly higher bacterial loads compared to E (ECW56), H (ECW568), and F (ECW58). Table S7. Slice test of least squares means for *X. euvesicatoria* at 6 dpi. Significant genotypic differences in bacterial populations persisted at 6 days post-inoculation (*p* < 0.0001). Figure S6. Tukey grouping of LS-means for bacterial populations in pepper genotypes inoculated with *Xanthomonas euvesicatoria* (Xe157) at 6 days post-inoculation. LS-means connected by the same bar are not significantly different based on Tukey’s HSD test (α = 0.05). Genotypes A(ECW), C(ECW6), D(ECW8), G (ECW68), B(ECW5), and F(ECW58) formed the highest grouping (red), indicating similar and elevated bacterial loads. Genotypes B(ECW5), F(ECW58), E(ECW56), and G(ECW68) also shared an intermediate grouping (blue), while genotype H (ECW568) formed a distinct group (green) with significantly lower bacterial population, suggesting enhanced resistance to *X. euvesicatoria*. Table S8. Slice test of least squares means for *X. euvesicatoria* at 9 dpi.No significant differences were observed among genotypes at 9 dpi (*p* = 0.2552). Figure S7. Tukey grouping of LS-means for bacterial populations in pepper genotypes inoculated with *Xanthomonas euvesicatoria* (Xe157) at 9 days post-inoculation. All genotypes fell within a single grouping (blue) based on Tukey’s HSD test (α = 0.05), indicating no statistically significant differences in bacterial populations among genotypes at this time point. Figure S8. Interaction plot of LS-means for Day × Genotype showing bacterial population dynamics (log CFU/cm^2^) in pepper lines inoculated with *Xanthomonas euvesicatoria*. LS-means were calculated across 3, 6, and 9 days post-inoculation with 95% confidence intervals. Each line represents a different genotype: A (ECW), B (ECW5), C (ECW6), D (ECW8), E (ECW56), F (ECW58), G (ECW68), and H (ECW568). Results indicate significant effects of time, genotype, and their interaction (*p* < 0.0001), with genotypes containing *bs5* alone or in combination (B, E, F, H) showing lower bacterial growth compared to other lines. Table S9: Type III tests of fixed effects for *Xanthomonas hortorum* pv. *gardneri* strain (Xhg) incubated at 20–25 °C over time and genotype. Strong effects for day, genotype, and their interaction (*p* < 0.0001) were observed, indicating a strong influence of both genotype and time on bacterial population dynamics. Table S10: Slice test of least squares means for genotypes inoculated with *Xhg* incubated at 20–25 °C at 3 dpi. Significant genotypic differences in bacterial populations were observed (*p* < 0.0001). Figure S9. Tukey’s HSD comparison of pepper genotypes inoculated with *Xanthomonas hortorum* pv. *gardneri* (*Xhg*) strain *Xg444* at 20–25 °C at 3 days post-inoculation. Genotypes H (ECW568) and E (ECW58) exhibited significantly lower bacterial populations compared to the susceptible genotype A (ECW), while genotypes D (ECW8), B (ECW5), and F (ECW56) showed intermediate levels. Genotypes G (ECW68), C (ECW6), and A (ECW) did not differ significantly from one another, indicating higher bacterial loads. Distinct letters denote statistically significant differences (*p* < 0.05). Table S11. Slice test of least squares means for genotypes inoculated with *Xhg* incubated at 20–25 °C at 6 dpi. Genotypic differences remained significant at 6 dpi (*p* < 0.0001). Figure S10. Tukey’s HSD comparison of pepper genotypes inoculated with *Xanthomonas hortorum* pv. *gardneri* (*Xhg)* at 20–25 °C at 6 days post-inoculation. Genotypes E (ECW58), H (ECW568), and F (ECW56) exhibited significantly lower bacterial loads compared to the susceptible genotype A (ECW). In contrast, genotypes B (ECW5), C (ECW6), D (ECW8), and G (ECW68) did not differ significantly from the susceptible control. Distinct letters denote statistically significant differences (*p* < 0.05). Table S12. Slice test of least squares means for genotypes inoculated with *Xhg* incubated at 20–25 °C at day 9. No significant differences among genotypes were observed at 9 dpi (*p* = 0.3888). Figure S11. Tukey’s HSD comparison of pepper genotypes inoculated with *Xanthomonas hortorum* pv. *gardneri* (*Xhg*) at 20–25 °C at 9 days post-inoculation. Genotypes E (ECW58) and H (ECW568) exhibited significantly lower bacterial populations than the susceptible genotype A (ECW). Genotypes B (ECW5), D (ECW8), and F (ECW56) showed intermediate levels, while genotypes G (ECW68), C (ECW6), and A (ECW) maintained the highest bacterial loads. Distinct letters denote statistically significant differences (*p* < 0.05). Figure S12. LS-Means plot with 95% confidence intervals for the interaction between pepper genotype and days post-inoculation following inoculation with *Xanthomonas hortorum* pv. *gardneri* (*Xhg*) at 20–25 °C. Genotype A (ECW) consistently exhibited the highest bacterial population across all time points. Genotypes E (ECW58) and H (ECW568) maintained the lowest bacterial loads by 9 dpi, with a significant divergence becoming evident over time. The trends suggest that stacking recessive resistance genes *bs5* and *bs8* confers enhanced and sustained resistance under low-temperature conditions. Table S13. Type III tests of fixed effects for *X. perforans* (Xp706) population in pepper genotypes across time. Significant effects were observed for day (*p* = 0.0002) and genotype (*p* < 0.0001). The day-by-genotype interaction approached significance (*p* = 0.0534), suggesting some genotype-specific temporal trends. Table S14. Slice test of least squares means for Xp706 at 3 dpi. Significant genotypic differences were found at 3 dpi (*p* < 0.0001). Figure S13. Tukey’s HSD comparison of pepper genotypes inoculated with Xanthomonas perforans strain Xp706 at 3 days post-inoculation (dpi). Genotypes A (ECW), C (ECW6), H (ECW568), and G (ECW58) grouped together with the highest mean bacterial populations. In contrast, genotype E (ECW5) exhibited the lowest bacterial load and was statistically distinct from most other genotypes, suggesting early effectiveness of the bs5 resistance gene against X. perforans. Distinct bars indicate statistically significant groupings (*p* < 0.05). Table S15. Slice test of least squares means for Xp706 at 6 dpi. Genotypic differences in bacterial populations remained significant at 6 dpi (*p* < 0.0001). Figure S14. Tukey’s HSD comparison of pepper genotypes inoculated with Xanthomonas perforans strain Xp706 at 6 days post-inoculation (dpi). Genotypes A (ECW), D (ECW56), and G (ECW58) exhibited the highest bacterial loads and grouped together as not significantly different. Genotype B (ECW6) remained intermediate, while genotypes E (ECW5), F (ECW568), and H (ECW8) displayed the lowest population estimates, with ECW5 and ECW568 showing statistically significant reductions in bacterial growth. Distinct bars indicate statistically significant groupings (*p* < 0.05). Figure S15. LS-Means analysis of pepper genotypes inoculated with *Xanthomonas perforans* strain Xp706 at 3 and 6 days post-inoculation (dpi), with 95% confidence limits. Genotypes A (ECW), C (ECW6), D (ECW8), and G (ECW68) exhibited higher LS-Means values, indicating elevated bacterial populations. In contrast, genotypes B (ECW5), E (ECW56), F (ECW58), and H (ECW568) showed consistently lower LS-Means, suggesting enhanced resistance to *X. perforans*. These results underscore the contribution of *bs5*—especially in combination with *bs6* or *bs8*—in suppressing bacterial proliferation. Table S16. Type III tests of fixed effects for *Xg444* populations incubated at 27–35 °C over time and genotype. All main effects and the day-by-genotype interaction were statistically significant (*p* ≤ 0.0050), suggesting variable resistance across genotypes and time points. Table S17. Slice test of least squares means for *Xg444* incubated at 27–35 °C at 3 dpi. Significant differences in bacterial populations were found among genotypes at 3 dpi (*p* < 0.0001). Figure S16. Tukey’s HSD comparison of pepper genotypes inoculated with *Xanthomonas hortorum* pv. *gardneri* (*Xhg*) strain *Xg444* and incubated at 27–35 °C at 3 days post-inoculation. Genotypes G (ECW68), C (ECW6), and A (ECW) exhibited the highest bacterial loads and were grouped in the top statistical subset. Intermediate bacterial levels were observed in D (ECW8), B (ECW5), and F (ECW58). The lowest bacterial populations were recorded in E (ECW56) and H (ECW568), both of which formed a separate statistical group, indicating significantly enhanced resistance. Distinct bars denote statistically significant differences (*p* < 0.05). Table S18. Slice test of least squares means for *Xg444* incubated at 27–35 °C at 6 dpi. Genotypic differences remained significant at 6 dpi (*p* < 0.0001). Figure S17. Tukey’s HSD comparison of pepper genotypes inoculated with *Xanthomonas hortorum* pv. *gardneri* (*Xhg*) strain *Xg444* and incubated at 27–35 °C at 6 days post-inoculation. Genotypes A (ECW), E (ECW56), D (ECW8), C (ECW6), B (ECW5), G (ECW68), and F (ECW58) formed a single statistical group with similar bacterial loads. Genotype H (ECW568) exhibited significantly reduced bacterial population levels and was statistically distinct from the rest, suggesting that pyramiding bs5, bs6, and bs8 enhances resistance under elevated temperature conditions. Distinct bars denote statistically significant differences (*p* < 0.05). Table S19. Slice test of least squares means for *Xg444* incubated at 27–35 °C at 9 dpi. No significant differences among genotypes were observed at 9 dpi (*p* = 0.3888). Figure S18. Tukey’s HSD comparison of pepper genotypes inoculated with *Xanthomonas hortorum* pv. *gardneri* (*Xhg*) strain *Xg444* and incubated at 27–35 °C at 9 days post-inoculation. Although ECW568 (H) exhibited the lowest numerical LS-mean estimate, all genotypes are grouped within the same statistical grouping, indicating no significant differences in bacterial population levels at this time point (*p* > 0.05). Distinct letters or bars indicate statistically significant differences when present. Figure S19. Line plot of least squares means (LS-Means) for the interaction between day and genotype, illustrating *Xg444* population dynamics in pepper genotypes inoculated and incubated at 27–35 °C. ECW568 (H) consistently exhibited the lowest LS-mean values across all time points, while ECW (A), ECW6 (F), and ECW56 (E) maintained higher bacterial levels by 9 dpi. Although overlapping 95% confidence intervals limited statistical significance, genotypes containing *bs5*, particularly in combination with other resistance genes (e.g., ECW58 and ECW568), showed a clear trend of reduced bacterial growth.

## References

[1] Jones J.B., Zitter T.A., Momol T.M., Miller S.A. (2016). Compendium of Tomato Diseases and Pests.

[2] Osdaghi E., Jones J.B., Sharma A., Goss E.M., Abrahamian P., Newberry E.A., Potnis N., Carvalho R., Choudhary M., Paret M.L. (2021). A centenary for bacterial spot of tomato and pepper. Mol. Plant Pathol..

[3] Bashan Y., Azaizeh M., Diab S., Yunis H., Okon Y. (1985). Crop loss of pepper plants artificially infected with *Xanthomonas campestris* pv. *vesicatoria* in relation to symptom expression. Crop Prot..

[4] Pohronezny K., Volin R.B. (1983). The effect of bacterial spot on yield and quality of fresh market tomatoes. HortScience.

[5] Jones J.B., Lacy G.H., Bouzar H., Stall R.E., Schaad N.W. (2004). Reclassification of the xanthomonads associated with bacterial spot disease of tomato and pepper. Syst. Appl. Microbiol..

[6] Potnis N., Timilsina S., Strayer A., Shantharaj D., Barak J.D., Paret M.L., Vallad G.E., Jones J.B. (2015). Bacterial spot of tomato and pepper: Diverse *Xanthomonas* species with a wide variety of virulence factors posing a worldwide challenge. Mol. Plant Pathol..

[7] Subedi A., Minsavage G.V., Jones J.B., Goss E.M., Roberts P.D. (2023). Exploring diversity of bacterial spot associated *Xanthomonas* population of pepper in southwest florida. Plant Dis..

[8] Horvath D.M., Stall R.E., Jones J.B., Pauly M.H., Vallad G.E., Dahlbeck D., Staskawicz B.J., Scott J.W. (2012). Transgenic resistance confers effective field level control of bacterial spot disease in tomato. PLoS ONE.

[9] Ritchie D. (2000). Bacterial Spot of Pepper and Tomato. Plant Health Instr..

[10] Rowell B., Jones R.T., Nesmith W., Satanek A., Snyder J.C. (2001). Bacterial spot resistance, yield, and quality of bell and specialty peppers. HortTechnology.

[11] Araújo E.R., Pereira R.C., Ferreira M.A.S.V., Café-Filho A.C., Moita A.W., Quezado-Duval A.M. (2011). Effect of temperature on pathogenicity components of tomato bacterial spot and competition between *Xanthomonas perforans* and *X. gardneri*. Acta Hortic..

[12] Jones J.B. (1986). Survival of *Xanthomonas campestris* pv. *vesicatoria* in Florida on tomato crop residue, weeds, seeds, and volunteer tomato plants. Phytopathology.

[13] Stall R.E., Jones J.B., Minsavage G.V. (2009). Durability of resistance in tomato and pepper to xanthomonads causing bacterial spot. Annu. Rev. Phytopathol..

[14] Sharma A., Minsavage G.V., Gill U.S., Hutton S.F., Jones J.B. (2022). identification and mapping of *bs8,* a novel locus conferring resistance to bacterial spot caused by *Xanthomonas gardneri*. Phytopathology.

[15] Gassmann W., Dahlbeck D., Chesnokova O., Minsavage G.V., Jones J.B., Staskawicz B.J. (2000). Molecular evolution of virulence in natural field strains of *Xanthomonas campestris* pv. *vesicatoria*. J. Bacteriol..

[16] Kousik C.S., Ritchie D.F. (1998). Response of bell pepper cultivars to bacterial spot pathogen races that individually overcome major resistance genes. Plant Dis..

[17] Jones J.B., Minsavage G.V., Roberts P.D., Johnson R.R., Kousik C.S., Subramanian S., Stall R.E. (2002). A non-hypersensitive resistance in pepper to the bacterial spot pathogen is associated with two recessive genes. Phytopathology.

[18] Vallejos C.E., Jones V., Stall R.E., Jones J.B., Minsavage G.V., Schultz D.C., Rodrigues R., Olsen L.E., Mazourek M. (2010). Characterization of Two Recessive Genes Controlling Resistance to All Races of Bacterial Spot in Peppers. Theor. Appl. Genet..

[19] Sharma A., Li J., Wente R., Minsavage G.V., Gill U.S., Ortega A., Vallejos C.E., Hart J.P., Staskawicz B.J., Mazourek M.R. (2023). Mapping of the *bs5* and *bs6* Non-Race-Specific Recessive Resistances against Bacterial Spot of Pepper. Front. Plant Sci..

[20] Schwartz A.R., Potnis N., Timilsina S., Wilson M., Patané J., Martins J., Minsavage G.V., Dahlbeck D., Akhunova A., Almeida N. (2015). Phylogenomics of *Xanthomonas* field strains infecting pepper and tomato reveals diversity in effector repertoires and identifies determinants of host specificity. Front. Microbiol..

[21] Madden L.V., Hughes G., Van Den Bosch F. (2007). The Study of Plant Disease Epidemics.

[22] Subedi A., Minsavage G.V., Roberts P.D., Goss E.M., Sharma A., Jones J.B. (2024). Insights into *bs5* resistance mechanisms in pepper against *Xanthomonas euvesicatoria* through transcriptome profiling. BMC Genom..

[23] Jones J.D.G., Dangl J.L. (2006). The Plant Immune System. Nature.

