# Supplementary material for: Pyramiding Recessive Resistance Genes Enhances Bacterial Leaf Spot Resistance in Peppers by Suppressing In Planta Bacterial Growth
Source: Plants (Basel). 2025 Aug 17;14(16):2559. doi: 10.3390/plants14162559 (PMC12389147; doi:10.3390/plants14162559)

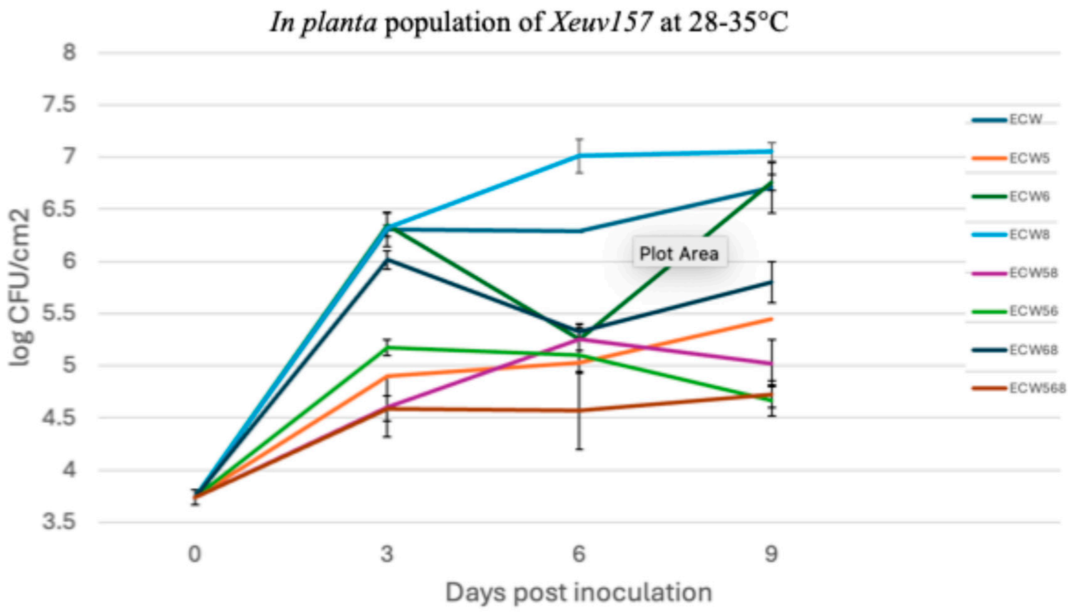

Supplementary Figure. S1. *In planta* growth of *Xanthomonas euvesicatoria* (strain Xe157, 10^5 CFU/ml) infiltrated in leaves of susceptible and resistant pepper lines over 9 dpi, incubated in greenhouse at 28–35°C (Experiment 2). Genotypes include ECW, Early CalWonder; ECW5 (*bs5*); ECW6 (*bs6*), ECW8 (*bs8*), ECW58 (*bs5*+*bs8*), ECW56 (*bs5*+*bs6*), ECW68 (*bs6*+ *bs8*), ECW568 (*bs5*+*bs6*+*bs8*). Each point represents the mean of three biological replicates (individual plants), and error bars indicate standard error (SE) across these replicates.

Supplementary Table S1: Area under the pathogen progress curve (AUPPC) for *Xanthomonas euvesicatoria* strain Xe157 in pepper genotypes, incubated in greenhouse at 28–35°C (Experiment 2). AUPPC was calculated from 0 to 9 days post-inoculation using the trapezoidal method. Genotypes include ECW (susceptible control), ECW5 (*bs5*), ECW6 (*bs6*), ECW8 (*bs8*), ECW56 (*bs5* + *bs6*), ECW58 (*bs5* + *bs8*), ECW68 (*bs6* + *bs8*), and ECW568 (*bs5* + *bs6* + *bs8*). Different letters indicate statistically significant differences among genotypes based on Student–Newman–Keuls (SNK) multiple comparisons at $\alpha = 0.05$.

Genotype	AUPPC (Exp 2)	SNK group
ECW568	40.19392	e
ECW58	42.58304	de
ECW56	42.71115	de
ECW5	43.57537	d
ECW68	48.33229	c
ECW6	50.5582	c
ECW	53.46997	b
ECW8	56.1993	a

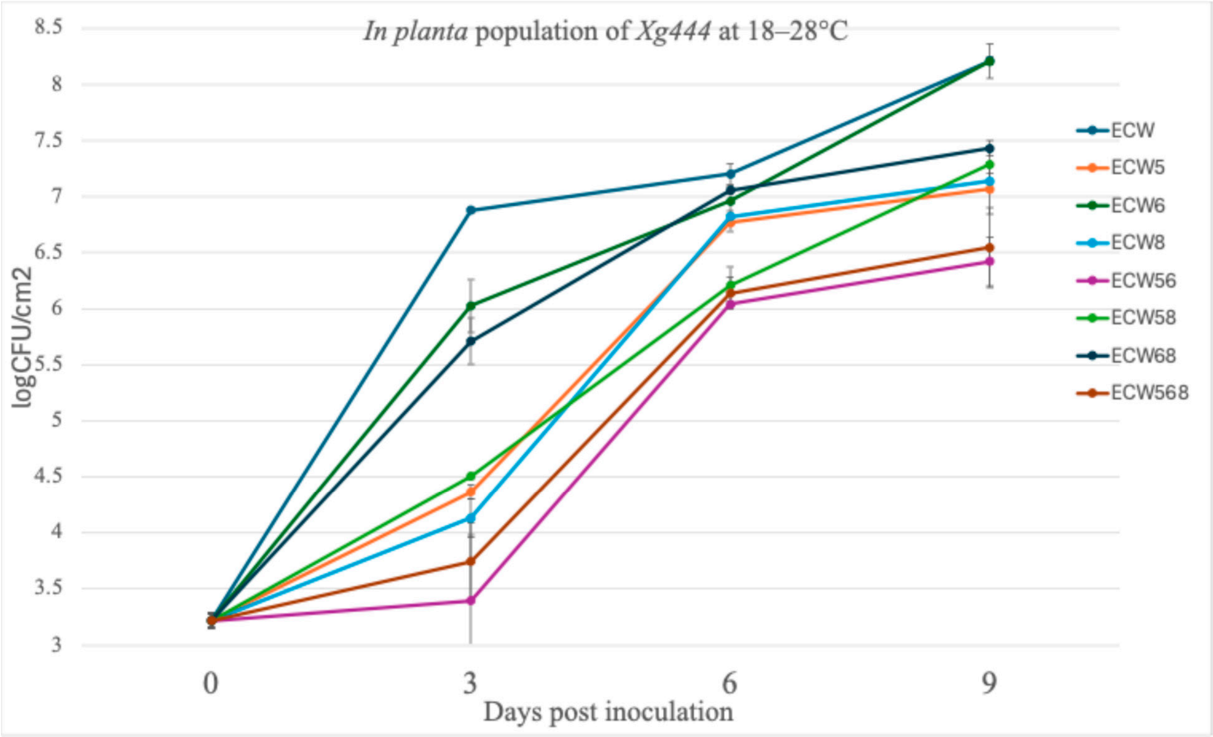

Supplementary Figure S2. *In planta* growth of *Xanthomonas hortorum* pv. *gardneri* (strain Xg444*,* 10^5 CFU/ml) infiltrated in leaves of susceptible and resistant pepper lines and incubated under greenhouse conditions ranging from 18–28°C for 0,3,6 and 9 days (Experiment 2). Genotypes include ECW, Early CalWonder; ECW5 (*bs5*); ECW6 (*bs6*), ECW8 (*bs8*), ECW58 (*bs5*+*bs8*), ECW56 (*bs5*+*bs6*), ECW68 (*bs6*+ *bs8*), ECW568 (*bs5*+*bs6*+*bs8*). Each point represents the mean of three biological replicates (individual plants), and error bars indicate standard error (SE) across these replicates.

Supplementary Table S2: Area under the pathogen progress curve (AUPPC) for *Xanthomonas hortorum* pv. gardneri strain Xg444 in pepper genotypes under 18–28°C incubation (Experiment 2). AUPPC was calculated from 0 to 9 days post-inoculation using the trapezoidal method. Genotypes include ECW, Early CalWonder; ECW5 (*bs5*); ECW6 (*bs6*), ECW8 (*bs8*), ECW58 (*bs5*+*bs8*), ECW56 (*bs5*+*bs6*), ECW68 (*bs6*+ *bs8*), ECW568 (*bs5*+*bs6*+*bs8*). Different letters indicate statistically significant differences among genotypes based on Student–Newman–Keuls (SNK) multiple comparisons at α = 0.05.

Genotype	AUPPC (Exp 2)	SNK group
ECW568	39.46055	e
ECW56	42.76482	d
ECW58	47.90051	c
ECW8	48.40376	c
ECW5	48.81529	c
ECW68	54.26971	b
ECW6	56.08611	b
ECW	59.38576	a

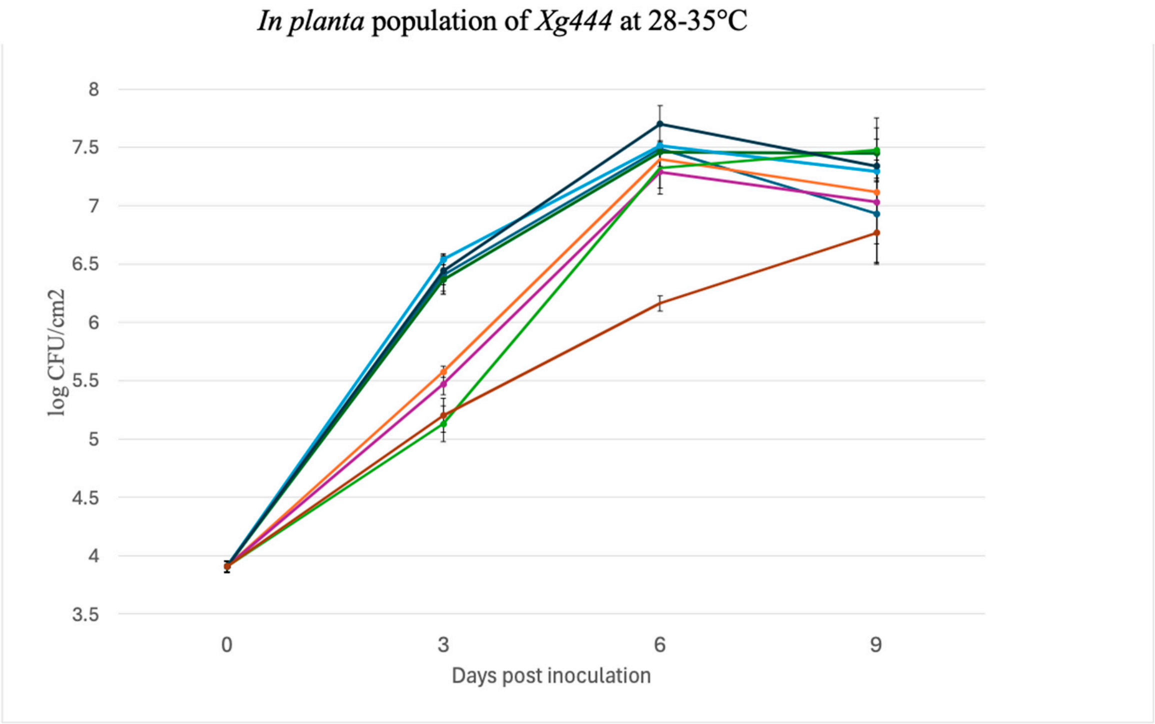

Supplementary Figure S3. *In planta* growth of *Xanthomonas hortorum* pv. *gardneri* (strain *Xg444,* 10^5 CFU/ml) infiltrated in leaves of susceptible and resistant pepper lines and incubated under greenhouse conditions ranging from 28–35°C for 0,3,6 and 9 days. Genotypes include ECW, Early CalWonder; ECW5 (*bs5*); ECW6 (*bs6*), ECW8 (*bs8*), ECW58 (*bs5*+*bs8*), ECW56 (*bs5*+*bs6*), ECW68 (*bs6*+ *bs8*), ECW568 (*bs5*+*bs6*+*bs8*). Each point represents the mean of three biological replicates (individual plants), and error bars indicate standard error (SE) across these replicates.

Supplementary Table S3: Area under the pathogen progress curve (AUPPC) for *Xanthomonas hortorum* pv. *gardneri* strain Xg444 in pepper genotypes incubated under greenhouse conditions ranging from 28–35°C (Experiment 2). AUPPC was calculated from 0 to 9 days post-inoculation using the trapezoidal method. Genotypes include ECW, Early CalWonder; ECW5 (*bs5*); ECW6 (*bs6*), ECW8 (*bs8*), ECW58 (*bs5+bs8*), ECW56 (*bs5+bs6*), ECW68 (*bs6+ bs8*), ECW568 (*bs5+bs6+bs8*).. Different letters indicate statistically significant differences among genotypes based on Student–Newman–Keuls (SNK) multiple comparisons at α = 0.05.

Genotype	AUPPC (Exp 2)	SNK group
ECW68	59.29981	a
ECW568	49.78913	c
ECW58	54.44248	b
ECW5	54.55787	b
ECW56	54.68982	b
ECW	57.94202	a
ECW6	58.51513	a
ECW8	58.96752	a

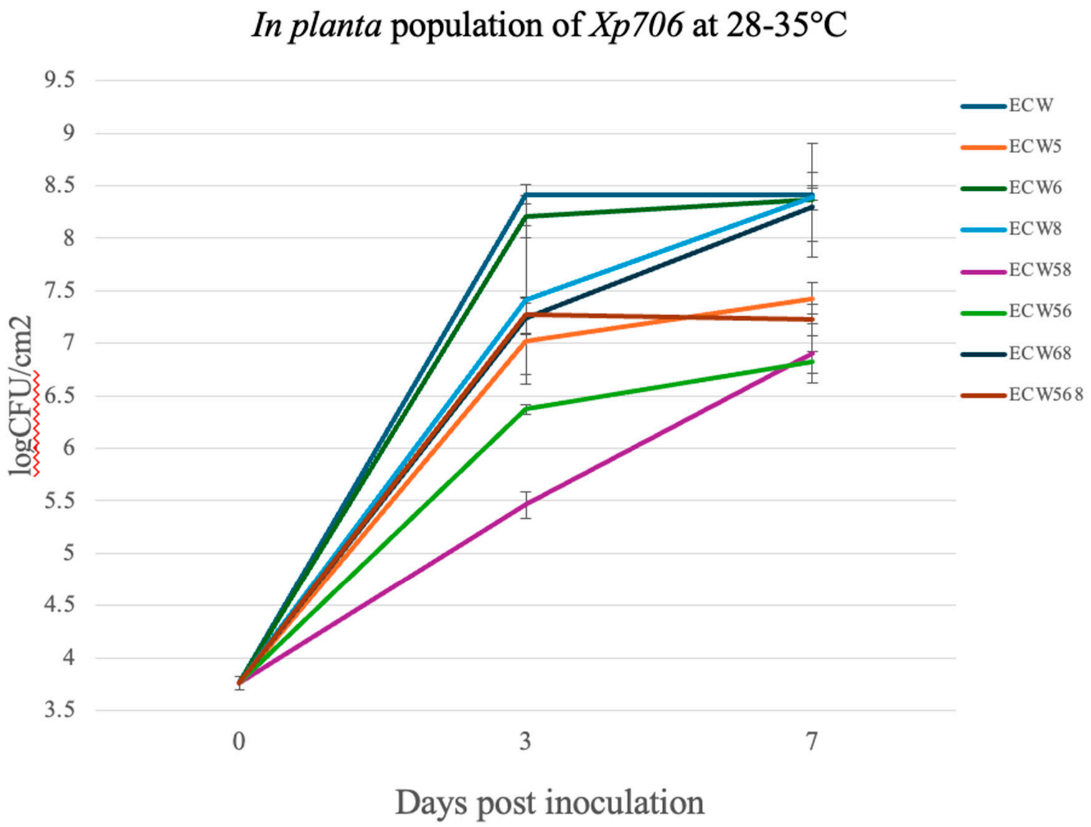

Supplementary Figure S4. *In planta* bacterial populations of *Xanthomonas perforans* strain Xp706 infiltrated with 10^5 CFU/mL in leaves of susceptible and resistant pepper genotypes at 0, 3, and incubated under greenhouse conditions ranging from 28–35°C (Experiment 2). Genotypes include ECW, Early CalWonder; ECW5 (*bs5*); ECW6 (*bs6*), ECW8 (*bs8*), ECW58 (*bs5*+*bs8*), ECW56 (*bs5*+*bs6*), ECW68 (*bs6*+ *bs8*), ECW568 (*bs5*+*bs6*+*bs8*). Each point represents the mean of three biological replicates (individual plants), and error bars indicate standard error (SE) across replicates.

Supplementary Table S4. Area under the pathogen progress curve (AUPPC) for *Xanthomonas perforans* strain Xp706 in pepper genotypes incubated at 28–35°C (Experiment 2). AUPPC was calculated from 0 to 7 days post-inoculation using the trapezoidal method. Genotypes include ECW, Early CalWonder; ECW5 (*bs5*); ECW6 (*bs6*), ECW8 (*bs8*), ECW58 (*bs5*+*bs8*), ECW56 (*bs5*+*bs6*), ECW68 (*bs6*+ *bs8*), ECW568 (*bs5*+*bs6*+*bs8*). Different letters indicate statistically significant differences among genotypes based on Student–Newman–Keuls (SNK) multiple comparisons at α = 0.05.

Genotype	AUPPC	SNK group
ECW56	38.29878	d
ECW58	39.11866	d
ECW5	44.50666	c
ECW568	45.35094	c
ECW8	46.54451	bc
ECW68	47.24861	bc
ECW	51.8907	a

Supplemental Table S5.Type III tests of fixed effects for *Xanthomonas euvesicatoria* (Xe157) population across pepper genotypes and time. All fixed effects—day, genotype, and their interaction—were highly significant (P < 0.0001), indicating strong temporal and genotypic differences in bacterial growth.

**Type III Tests of Fixed Effects**				
**Effect**	**Num DF**	**Den DF**	**F Value**	**Pr > F**
**Day**	2	15	1007.07	<.0001
**Genotypes**	7	16	11.75	<.0001
**Day*Genotypes**	14	18.08	15.13	<.0001

Supplemental Table S6. Slice test of least squares means for *X. euvesicatoria* at 3 dpi. Significant differences among genotypes were detected at 3 days post-inoculation (P < 0.0001).

**F Test for Day*Genotypes Least Squares Means Slice**				
**Slice**	**Num DF**	**Den DF**	**F Value**	**Pr > F**
**Day 3**	7	16	53.09	<.0001

Supplemental Figure S5. Tukey grouping of least square means for bacterial populations in pepper genotypes inoculated with *Xanthomonas euvesicatoria* (Xe157) at 3 days post-inoculation.LS-means estimates (log CFU/cm²) are grouped using Tukey's HSD test (α = 0.05). Bars sharing the same color indicate no significant difference in bacterial population levels. Genotypes D (ECW8), G (ECW68), A (ECW), and C(ECW6) exhibited significantly higher bacterial loads compared to E (ECW56), H (ECW568), and F (ECW58).

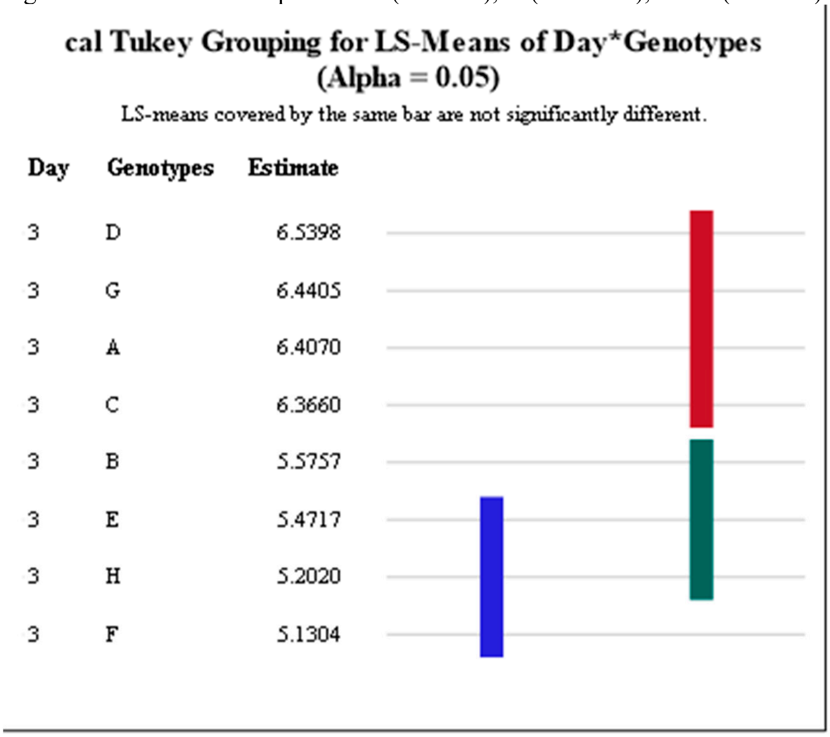

Supplemental Table S7. Slice test of least squares means for *X. euvesicatoria* at 6 dpi. Significant genotypic differences in bacterial populations persisted at 6 days post-inoculation (P < 0.0001).

F Test for Day*Genotypes Least Squares Means Slice				
**Slice**	**Num DF**	**Den DF**	**F Value**	**Pr > F**
**Day 6**	7	16	35.59	<.0001

Supplementary Figure S6. Tukey grouping of LS-means for bacterial populations in pepper genotypes inoculated with *Xanthomonas euvesicatoria (*Xe157*)* at 6 days post-inoculation. LS-means connected by the same bar are not significantly different based on Tukey's HSD test (α = 0.05). Genotypes A(ECW), C(ECW6), D(ECW8), G (ECW68), B(ECW5), and F(ECW58) formed the highest grouping (red), indicating similar and elevated bacterial loads. Genotypes B(ECW5), F(ECW58), E(ECW56), and G(ECW68) also shared an intermediate grouping (blue), while genotype H (ECW568) formed a distinct group (green) with significantly lower bacterial population, suggesting enhanced resistance to *X. euvesicatoria*.

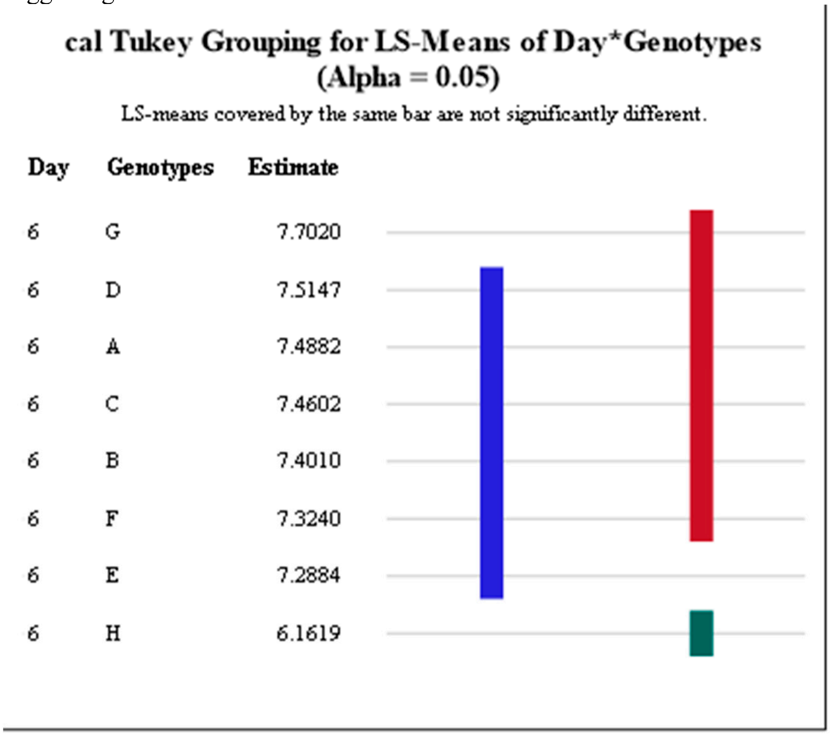

Supplemental Table S8.Slice test of least squares means for *X. euvesicatoria* at 9 dpi.No significant differences were observed among genotypes at 9 dpi (P = 0.2552).

F Test for Day*Genotypes Least Squares Means Slice				
**Slice**	**Num DF**	**Den DF**	**F Value**	**Pr > F**
**Day 9**	7	16	1.45	0.2552

Supplemental Figure S7. Tukey grouping of LS-means for bacterial populations in pepper genotypes inoculated with *Xanthomonas euvesicatoria* (Xe157) at 9 days post-inoculation. All genotypes fell within a single grouping (blue) based on Tukey's HSD test (α = 0.05), indicating no statistically significant differences in bacterial populations among genotypes at this time point.

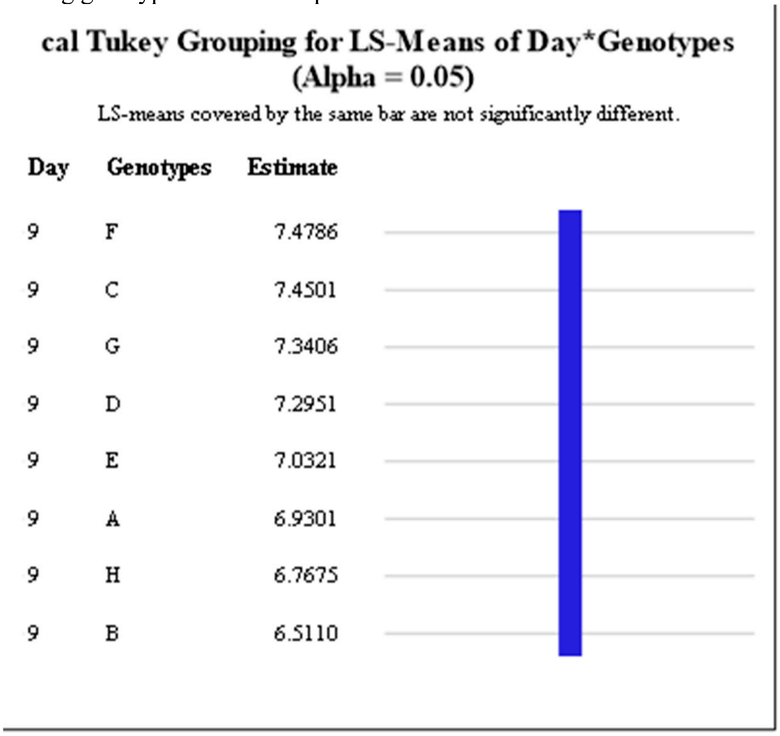

Supplementary Figure S8. Interaction plot of LS-means for Day × Genotype showing bacterial population dynamics (log CFU/cm²) in pepper lines inoculated with *Xanthomonas euvesicatoria*. LS-means were calculated across 3, 6, and 9 days post-inoculation with 95% confidence intervals. Each line represents a different genotype: A (ECW), B (ECW5), C (ECW6), D (ECW8), E (ECW56), F (ECW58), G (ECW68), and H (ECW568). Results indicate significant effects of time, genotype, and their interaction ($p < 0.0001$), with genotypes containing *bs5* alone or in combination (B, E, F, H) showing lower bacterial growth compared to other lines.

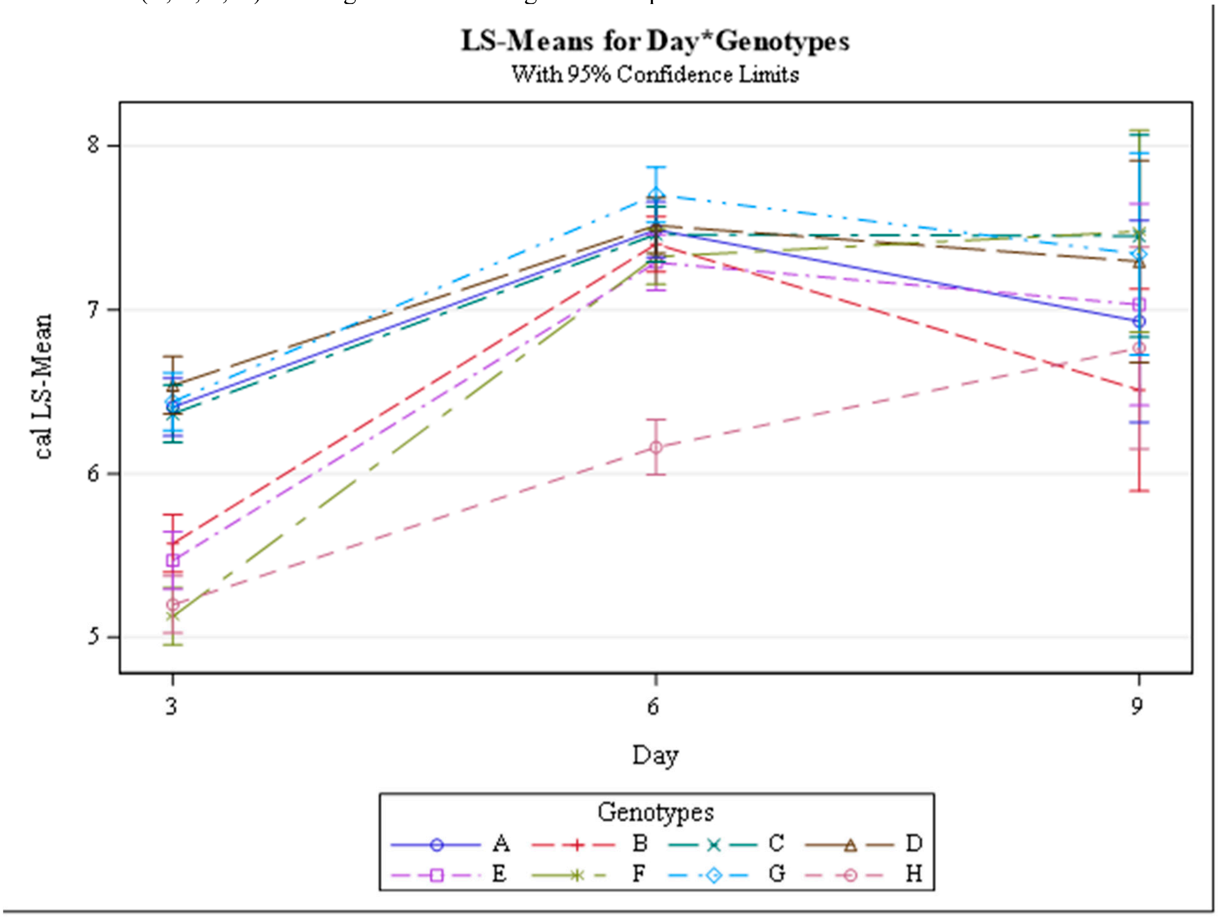

Supplementary Table S9: Type III tests of fixed effects for *Xanthomonas hortorum* pv. *gardneri* strain (Xhg) incubated at 20-25°C over time and genotype. Strong effects for day, genotype, and their interaction ($P < 0.0001$) were observed, indicating a strong influence of both genotype and time on bacterial population dynamics.

Type III Tests of Fixed Effects

**Effect**	**Num DF**	**Den DF**	**F Value**	**Pr > F**
**Day**	2	15	607.83	<.0001
**Genotypes**	7	16	78.06	<.0001
**Day*Genotypes**	14	18.08	9.34	<.0001

Supplementary Table S10: Slice test of least squares means for genotypes inoculated with *Xhg* incubated at 20-25°C at 3 dpi. Significant genotypic differences in bacterial populations were observed (P < 0.0001).

F Test for Day*Genotypes Least Squares Means Slice				
**Slice**	**Num DF**	**Den DF**	**F Value**	**Pr > F**
**Day 3**	7	16	42.45	<.0001

Supplemental Figure S9. Tukey's HSD comparison of pepper genotypes inoculated with *Xanthomonas hortorum* pv. *gardneri* (*Xhg*) strain Xg444 at 20–25°C at 3 days post-inoculation. Genotypes H (ECW568) and E (ECW58) exhibited significantly lower bacterial populations compared to the susceptible genotype A (ECW), while genotypes D (ECW8), B (ECW5), and F (ECW56) showed intermediate levels. Genotypes G (ECW68), C (ECW6), and A (ECW) did not differ significantly from one another, indicating higher bacterial loads. Distinct letters denote statistically significant differences (P < 0.05).

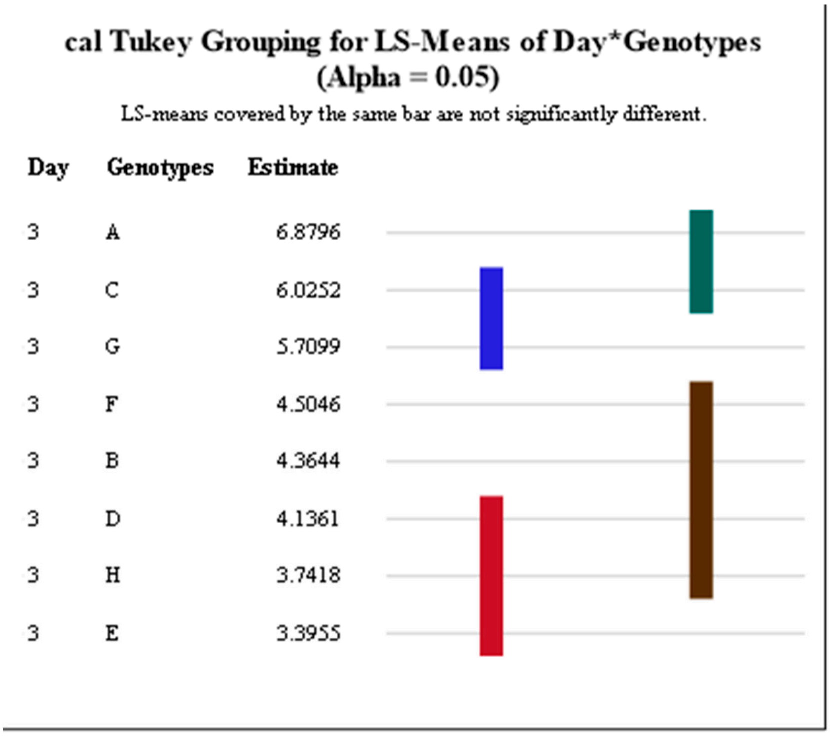

Supplemental Table S11. Slice test of least squares means for genotypes inoculated with *Xhg* incubated at 20-25°C at 6 dpi. Genotypic differences remained significant at 6 dpi ($P < 0.0001$).

F Test for Day*Genotypes Least Squares Means Slice				
**Slice**	**Num DF**	**Den DF**	**F Value**	**Pr > F**
**Day 6**	7	16	43.47	<.0001

Supplemental Figure S10. Tukey's HSD comparison of pepper genotypes inoculated with *Xanthomonas hortorum* pv. *gardneri* (*Xhg)* at 20–25°C at 6 days post-inoculation. Genotypes E (ECW58), H (ECW568), and F (ECW56) exhibited significantly lower bacterial loads compared to the susceptible genotype A (ECW). In contrast, genotypes B (ECW5), C (ECW6), D (ECW8), and G (ECW68) did not differ significantly from the susceptible control. Distinct letters denote statistically significant differences ($P < 0.05$).

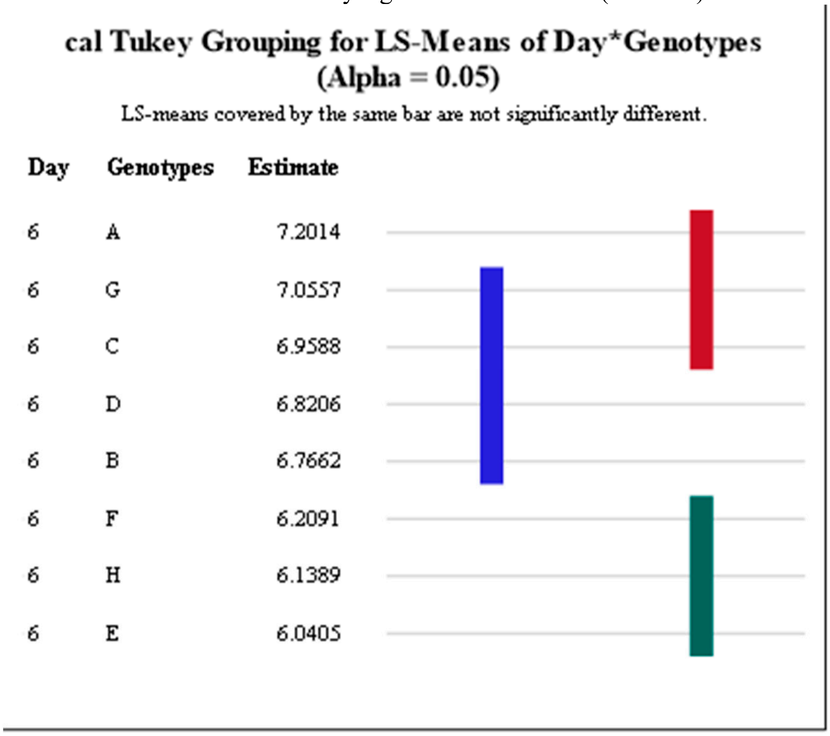

Supplemental Table S12. Slice test of least squares means for genotypes inoculated with *Xhg* incubated at 20-25°C at day 9.No significant differences among genotypes were observed at 9 dpi ($P = 0.3888$).

F Test for Day*Genotypes Least Squares Means Slice				
**Slice**	**Num DF**	**Den DF**	**F Value**	**Pr > F**
**Day 9**	7	16	25.32	<.0001

Supplemental Figure S11. Tukey’s HSD comparison of pepper genotypes inoculated with *Xanthomonas hortorum* pv. *gardneri* (*Xhg*) at 20–25°C at 9 days post-inoculation. Genotypes E (ECW58) and H (ECW568) exhibited significantly lower bacterial populations than the susceptible genotype A (ECW). Genotypes B (ECW5), D (ECW8), and F (ECW56) showed intermediate levels, while genotypes G (ECW68), C (ECW6), and A (ECW) maintained the highest bacterial loads. Distinct letters denote statistically significant differences (P < 0.05).

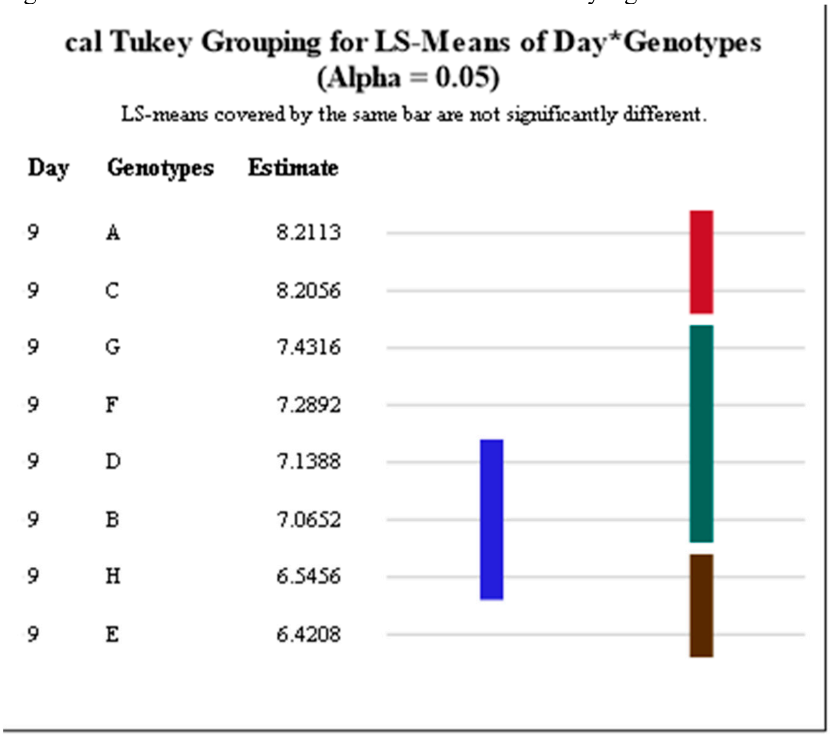

Supplemental Figure S12. LS-Means plot with 95% confidence intervals for the interaction between pepper genotype and days post-inoculation following inoculation with *Xanthomonas hortorum* pv. *gardneri* (*Xhg*) at 20–25°C. Genotype A (ECW) consistently exhibited the highest bacterial population across all time points. Genotypes E (ECW58) and H (ECW568) maintained the lowest bacterial loads by 9 dpi, with a significant divergence becoming evident over time. The trends suggest that stacking recessive resistance genes *bs5* and *bs8* confers enhanced and sustained resistance under low-temperature conditions.

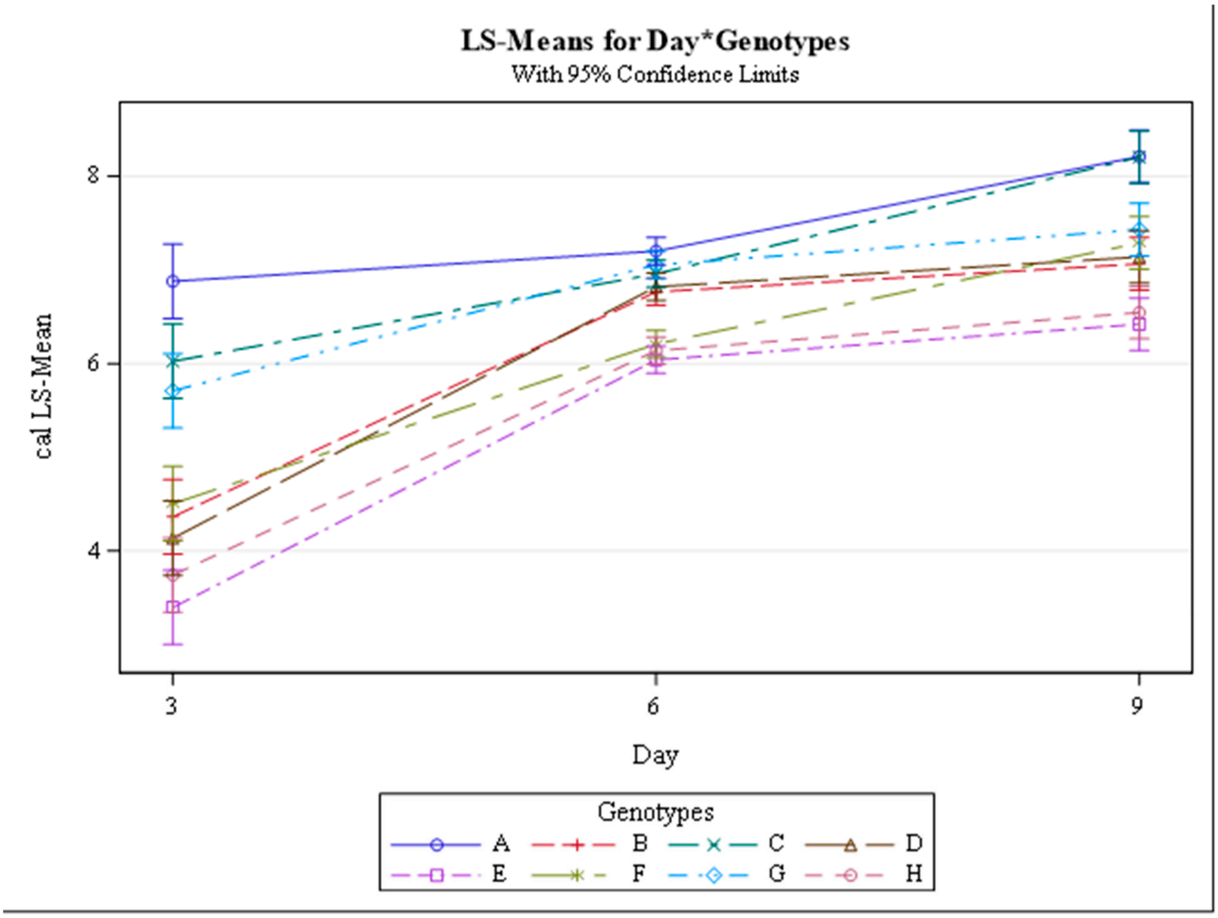

Supplemental Table S13.Type III tests of fixed effects for *X. perforans* (Xp706) population in pepper genotypes across time. Significant effects were observed for day ($P = 0.0002$) and genotype ($P < 0.0001$). The day-by-genotype interaction approached significance ($P = 0.0534$), suggesting some genotype-specific temporal trends.

Type III Tests of Fixed Effects				
**Effect**	**Num DF**	**Den DF**	**F Value**	**Pr > F**
**Day**	1	16	23.33	0.0002
**Genotypes**	7	16	21.06	<.0001
**Day*Genotypes**	7	16	2.61	0.0534

Supplemental Table S14.Slice test of least squares means for Xp706 at 3 dpi. Significant genotypic differences were found at 3 dpi ($P < 0.0001$).

F Test for Day*Genotypes Least Squares Means Slice				
**Slice**	**Num DF**	**Den DF**	**F Value**	**Pr > F**
**Day 3**	7	16	10.03	<.0001

Supplemental Figure S13. Tukey's HSD comparison of pepper genotypes inoculated with Xanthomonas perforans strain Xp706 at 3 days post-inoculation (dpi). Genotypes A (ECW), C (ECW6), H (ECW568), and G (ECW58) grouped together with the highest mean bacterial populations. In contrast, genotype E (ECW5) exhibited the lowest bacterial load and was statistically distinct from most other genotypes, suggesting early effectiveness of the bs5 resistance gene against X. perforans. Distinct bars indicate statistically significant groupings (P < 0.05).

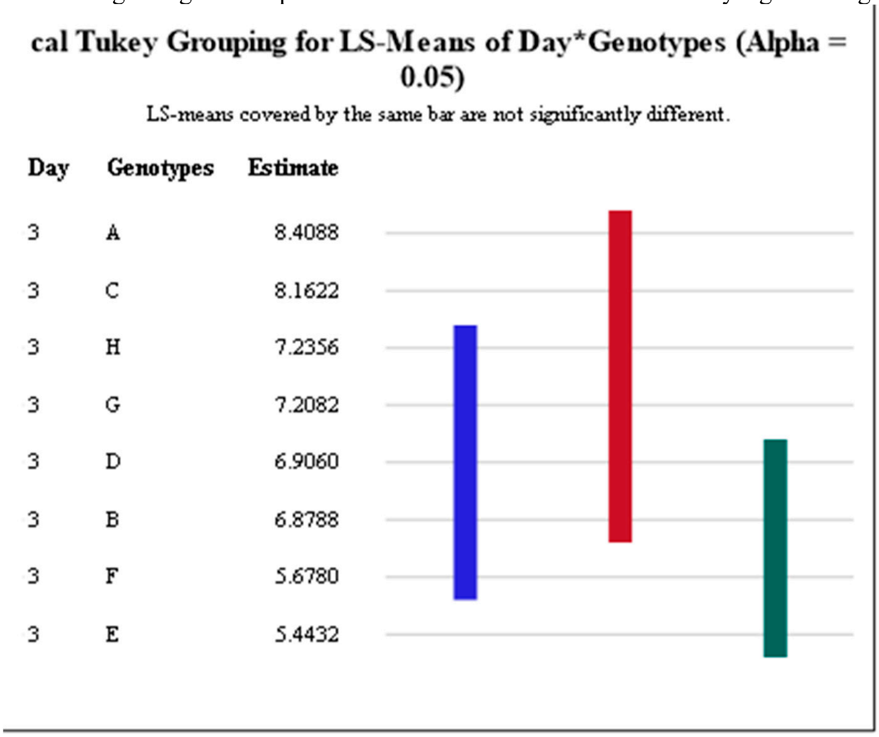

Supplemental Table S15.Slice test of least squares means for Xp706 at 6 dpi. Genotypic differences in bacterial populations remained significant at 6 dpi (P < 0.0001).

F Test for Day*Genotypes Least Squares Means Slice				
**Slice**	**Num DF**	**Den DF**	**F Value**	**Pr > F**
**Day 6**	7	16	13.78	<.0001

Supplemental Figure S14. Tukey's HSD comparison of pepper genotypes inoculated with Xanthomonas perforans strain Xp706 at 6 days post-inoculation (dpi). Genotypes A (ECW), D (ECW56), and G (ECW58) exhibited the highest bacterial loads and grouped together as not significantly different. Genotype B (ECW6) remained intermediate, while genotypes E (ECW5), F (ECW568), and H (ECW8) displayed the lowest population estimates, with ECW5 and ECW568 showing statistically significant reductions in bacterial growth. Distinct bars indicate statistically significant groupings ($P < 0.05$).

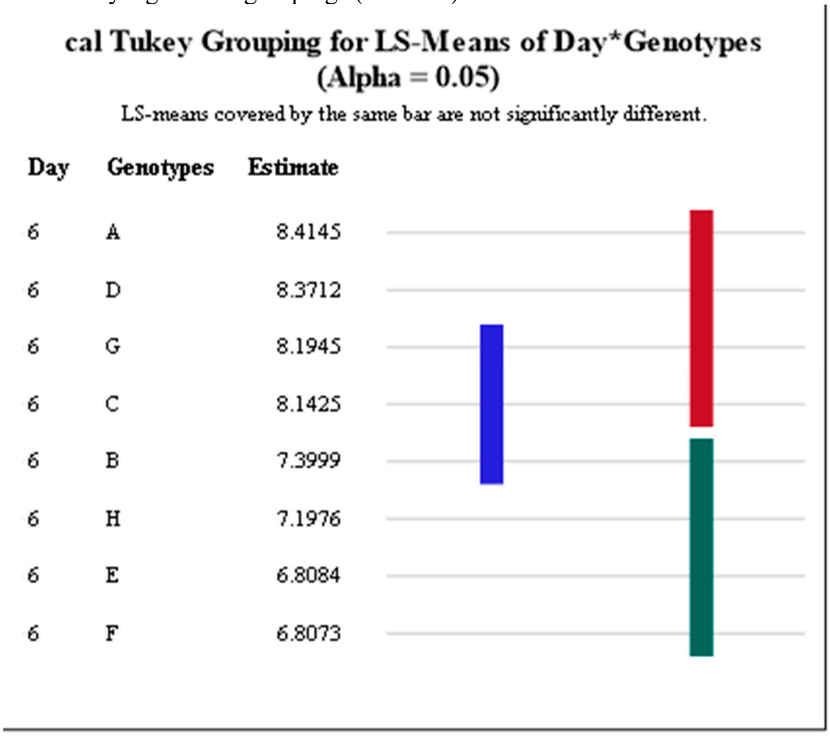

Supplemental Figure S15. LS-Means analysis of pepper genotypes inoculated with *Xanthomonas perforans* strain Xp706 at 3 and 6 days post-inoculation (dpi), with 95% confidence limits. Genotypes A (ECW), C (ECW6), D (ECW8), and G (ECW68) exhibited higher LS-Means values, indicating elevated bacterial populations. In contrast, genotypes B (ECW5), E (ECW56), F (ECW58), and H (ECW568) showed consistently lower LS-Means, suggesting enhanced resistance to *X. perforans*. These results underscore the contribution of bs5—especially in combination with bs6 or bs8—in suppressing bacterial proliferation.

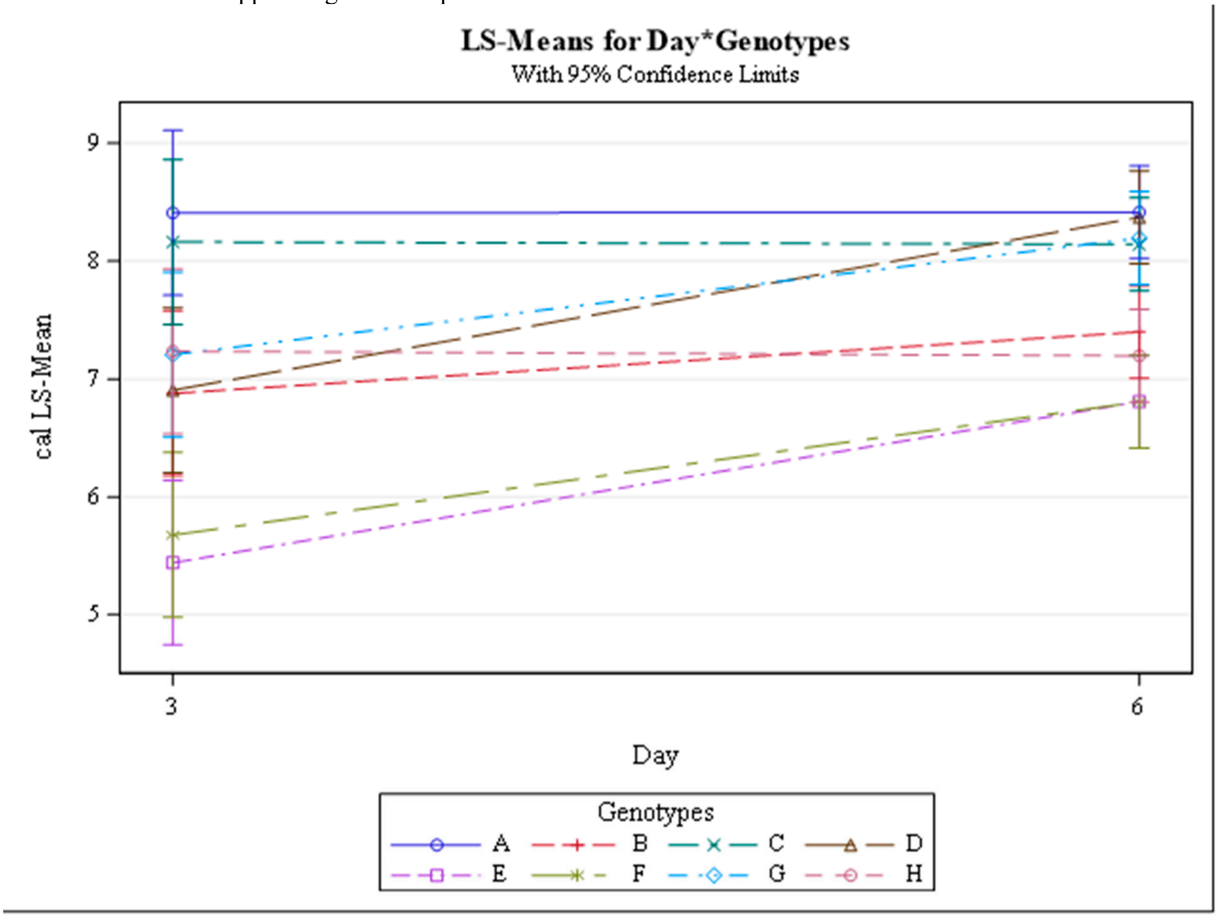

Supplemental Table S16. Type III tests of fixed effects for *X. hortorum pv. gardneri* (Xg444) populations incubated at 27-35° C over time and genotype. All main effects and the day-by-genotype interaction were statistically significant (P ≤ 0.0050), suggesting variable resistance across genotypes and time points.

Type III Tests of Fixed Effects				
**Effect**	**Num DF**	**Den DF**	**F Value**	**Pr > F**
**Day**	2	15	86.71	<.0001
**Genotypes**	7	16	6.34	0.0011
**Day*Genotypes**	14	18.08	3.72	0.0050

Supplemental Table S17.Slice test of least squares means for Xg444 incubated at 27-35° C at 3 dpi. Significant differences in bacterial populations were found among genotypes at 3 dpi (P < 0.0001).

F Test for Day*Genotypes Least Squares Means Slice				
**Slice**	**Num DF**	**Den DF**	**F Value**	**Pr > F**
**Day 3**	7	16	11.84	<.0001

Supplemental Figure S16. Tukey's HSD comparison of pepper genotypes inoculated with *Xanthomonas hortorum* pv. *gardneri* (*Xhg*) strain Xg444 and incubated at 27–35°C at 3 days post-inoculation. Genotypes G (ECW68), C (ECW6), and A (ECW) exhibited the highest bacterial loads and were grouped in the top statistical subset. Intermediate bacterial levels were observed in D (ECW8), B (ECW5), and F (ECW58). The lowest bacterial populations were recorded in E (ECW56) and H (ECW568), both of which formed a separate statistical group, indicating significantly enhanced resistance. Distinct bars denote statistically significant differences (P < 0.05).

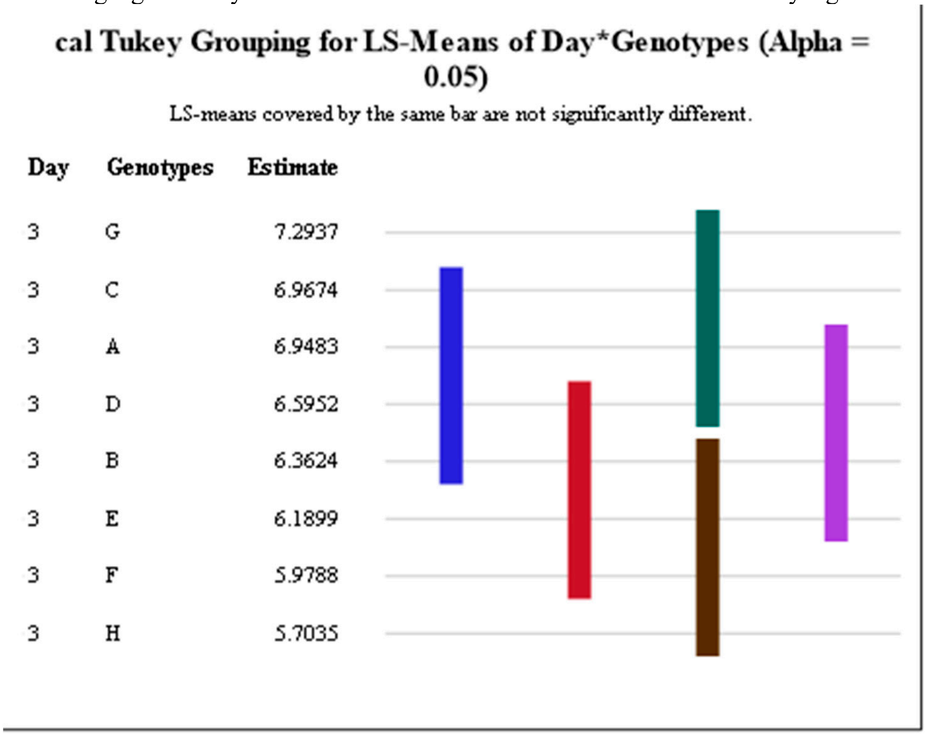

Supplemental Table S18.Slice test of least squares means for Xg444 incubated at 27-35° C at 6 dpi. Genotypic differences remained significant at 6 dpi (P < 0.0001).

F Test for Day*Genotypes Least Squares Means Slice				
**Slice**	**Num DF**	**Den DF**	**F Value**	**Pr > F**
**Day 6**	7	16	14.71	<.0001

Supplemental Figure S17. Tukey's HSD comparison of pepper genotypes inoculated with *Xanthomonas hortorum* pv. *gardneri* (*Xhg*) strain Xg444 and incubated at 27–35°C at 6 days post-inoculation. Genotypes A (ECW), E (ECW56), D (ECW8), C (ECW6), B (ECW5), G (ECW68), and F (ECW58) formed a single statistical group with similar bacterial loads. Genotype H (ECW568) exhibited significantly reduced bacterial population levels and was statistically distinct from the rest, suggesting that pyramiding bs5, bs6, and bs8 enhances resistance under elevated temperature conditions. Distinct bars denote statistically significant differences (P < 0.05).

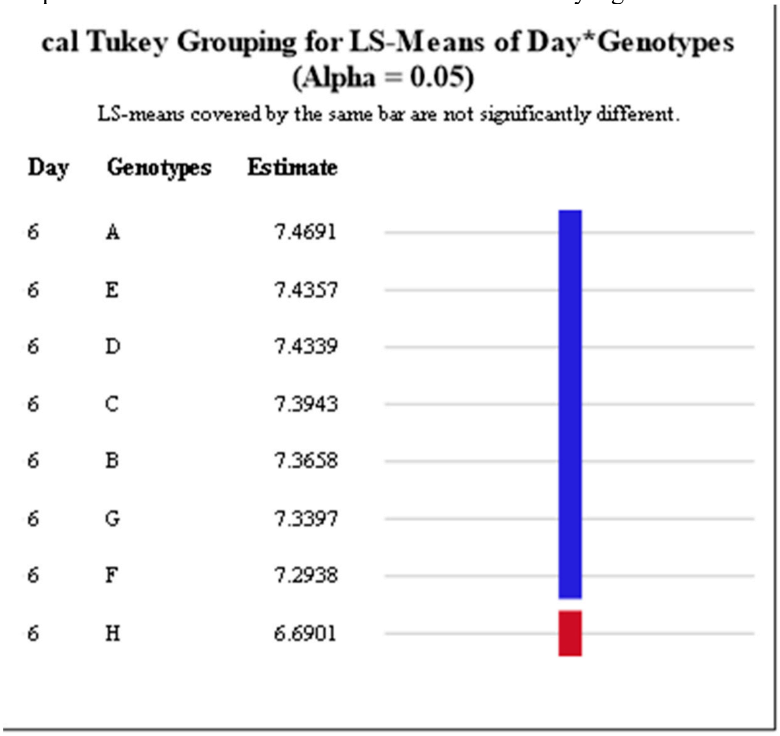

Supplemental Table S19.Slice test of least squares means for Xg444 incubated at 27-35°C at 9 dpi. No significant differences among genotypes were observed at 9 dpi (P = 0.3888).

F Test for Day*Genotypes Least Squares Means Slice				
**Slice**	**Num DF**	**Den DF**	**F Value**	**Pr > F**
**Day 9**	7	16	1.14	0.3888

Supplemental Figure S18. Tukey's HSD comparison of pepper genotypes inoculated with *Xanthomonas hortorum* pv. *gardneri* (*Xhg*) strain Xg444 and incubated at 27–35 °C at 9 days post-inoculation. Although ECW568 (H) exhibited the lowest numerical LS-mean estimate, all genotypes are grouped within the same statistical grouping, indicating no significant differences in bacterial population levels at this time point (P > 0.05). Distinct letters or bars indicate statistically significant differences when present.

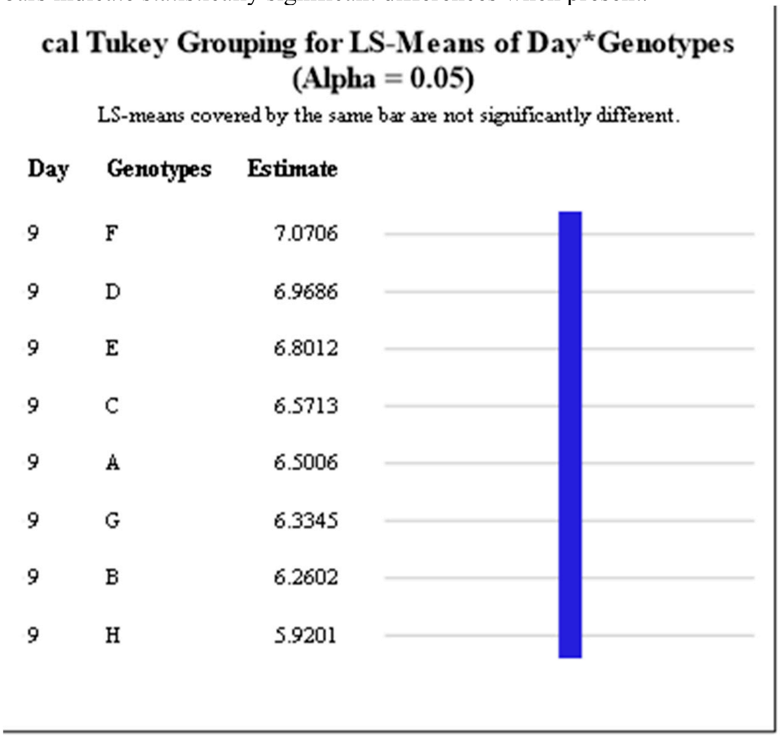

Supplemental Figure S19. Line plot of least squares means (LS-Means) for the interaction between day and genotype, illustrating *Xanthomonas hortorum* pv. *gardneri* (*Xhg*) population dynamics in pepper genotypes inoculated and incubated at 27–35 °C. ECW568 (H) consistently exhibited the lowest LS-mean values across all time points, while ECW (A), ECW6 (F), and ECW56 (E) maintained higher bacterial levels by 9 dpi. Although overlapping 95% confidence intervals limited statistical significance, genotypes containing *bs5*, particularly in combination with other resistance genes (e.g., ECW58 and ECW568), showed a clear trend of reduced bacterial growth.

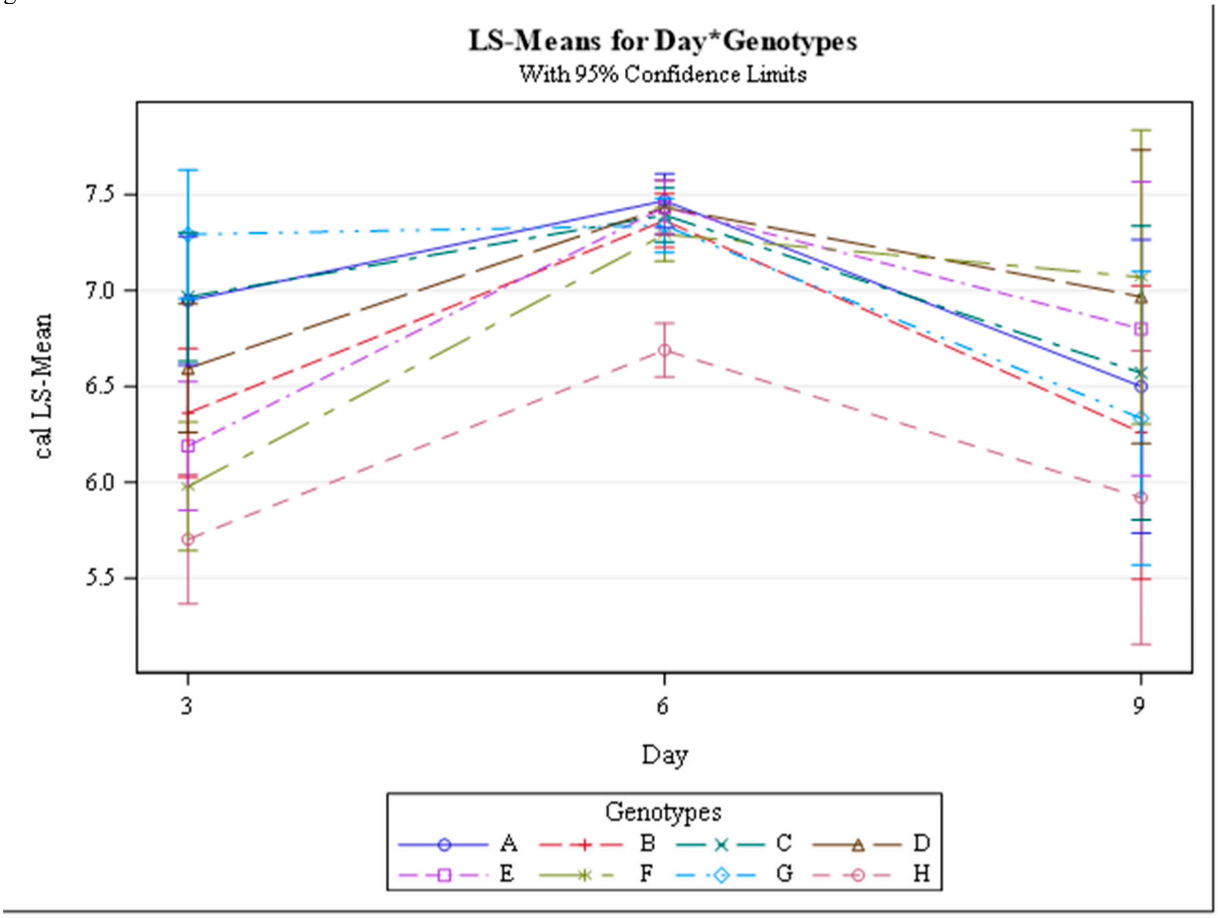

Supplement: Supplementary file 1 [file plants-14-02559-s001.zip › plants-3706708-supplementary.pdf]
